# Anti-Inflammatory and Antioxidant Pyrrolo[3,4-*d*]pyridazinone Derivatives Interact with DNA and Bind to Plasma Proteins—Spectroscopic and In Silico Studies

**DOI:** 10.3390/ijms25031784

**Published:** 2024-02-01

**Authors:** Aleksandra Kotynia, Edward Krzyżak, Julia Żądło, Maja Witczak, Łukasz Szczukowski, Jakub Mucha, Piotr Świątek, Aleksandra Marciniak

**Affiliations:** 1 Department of Basic Chemical Sciences, Faculty of Pharmacy, Wroclaw Medical University, Borowska 211a, 50-556 Wrocław, Poland; edward.krzyzak@umw.edu.pl; 2 “Biomolecule” Student Science Club, Department of Basic Chemical Sciences, Faculty of Pharmacy, Wroclaw Medical University, Borowska 211a, 50-556 Wrocław, Poland; julia.zadlo@student.umw.edu.pl (J.Ż.); maja.witczak@student.umw.edu.pl (M.W.); 3 Department of Medicinal Chemistry, Faculty of Pharmacy, Wroclaw Medical University, Borowska 211, 50-556 Wrocław, Poland; lukasz.szczukowski@umw.edu.pl (Ł.S.); piotr.swiatek@umw.edu.pl (P.Ś.); 4 Faculty of Pharmacy, Wroclaw Medical University, Borowska 211, 50-556 Wrocław, Poland; kubamucha14@gmail.com

**Keywords:** CD spectroscopy, DNA, fluorescence spectroscopy, molecular modelling, plasma proteins, pyridazinone derivatives

## Abstract

From the point of view of the search for new pharmaceuticals, pyridazinone derivatives are a very promising group of compounds. In our previous works, we have proved that newly synthesized ligands from this group have desirable biological and pharmacokinetic properties. Therefore, we decided to continue the research evaluating the activity of pyrrolo[3,4-*d*pyridazinone derivatives. In this work, we focused on the interactions of five pyridazinone derivatives with the following biomolecules: DNA and two plasma proteins: orosomucoid and gamma globulin. Using several of spectroscopic methods, such as UV-Vis, CD, and fluorescence spectroscopy, we proved that the tested compounds form stable complexes with all biomacromolecules selected for analysis. These findings were also confirmed by the results obtained by molecular modeling. All tested pyridazinone derivatives bind to the ctDNA molecule via groove binding mechanisms. All these molecules can also be bound and transported by the tested plasma proteins; however, the stability of the complexes formed is lower than those formed with serum albumin.

## 1. Introduction

Mono- and bi(hetero)cyclic pyridazinone derivatives have been an interesting subject of research in medicine and pharmacy for many years. Pyridazinone ring is present in compounds that have a wide spectrum of desirable properties, including anti-inflammatory, analgesic, and anticancer. This group of compounds also has cardiovascular, antinociceptive, antidiabetic, anti-asthmatic, anticonvulsant, and antidepressant activities [1,2,3,4,5].

**Figure 1:**
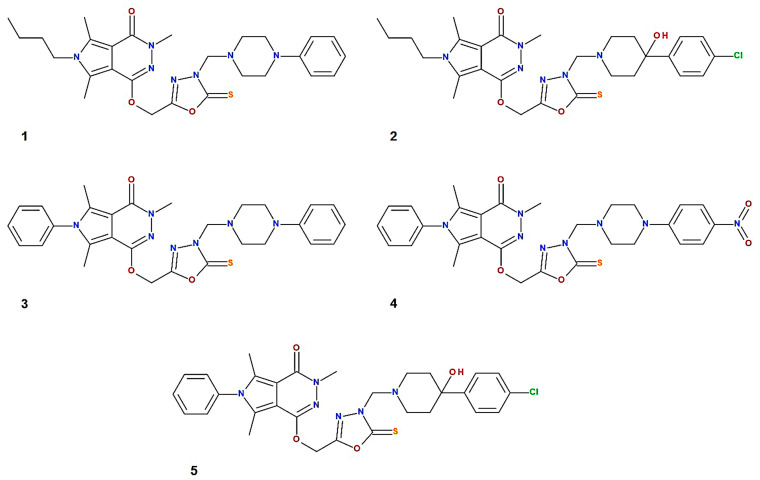
The structures of analyzed pyrrolo[3,4-*d*]pyridazinone derivatives **1**, **2**, **3**, **4**, and **5**.

In our previous study, we described the synthesis and biological evaluation of new fourteen pyrrolo[3,4-*d*]pyridazinone derivatives [1]. The results obtained were promising and showed the antioxidant and anti-inflammatory effects of the tested compounds. All of them also formed stable complexes with the serum albumin molecule. Subsequent studies confirmed the anti-inflammatory and analgesic effects of these derivatives [6,7,8]. For this reason, we decided to continue research on this group of compounds. In this study, we have chosen five pyridazinone derivatives, named, respectively, with consecutive numbers **1**–**5** (Figure 1). As part of this work, compounds that showed the best in vitro properties, based on the results of MCDA (multiple criteria decision analysis), were further analyzed [1].

The DNA double helix is a molecular target for many drugs and drug candidates. Often, to cure a disease or limit its effects, it is necessary to inhibit or change DNA function. The interaction of small molecules with this macromolecule leads to the desired effect [9,10]. Many anticancer drugs work this way. For this reason, testing the interaction with DNA in the case of new substances is extremely important from the point of view of designing new pharmaceuticals. Many compounds derived from pyridazinone that have anticancer properties have been described in the literature so far [5]. On the other hand, the analysis of the interaction of potential drugs with DNA can help determine their toxicity. Drugs that are toxic to the human body may have a significant impact on the structure of the DNA molecule and permanently and strongly deform or damage it. Therefore, testing compounds **1**–**5** for their interaction with the DNA molecule seems justified. Small ligands can bind to the DNA molecule by forming covalent bonds or by noncovalent interactions. In the case of the second of the mentioned methods of interaction, three basic mechanisms can be distinguished: electrostatic interaction, intercalation, and groove binding (minor and major) [10]. Often, a mixed mechanism of impact can be observed. The use of a number of spectroscopic methods combined with molecular modeling studies allow to determine which mechanism is dominant.

It is well known that the analysis of the interaction of new compounds with plasma proteins is important in the search for new pharmaceuticals. The method and strength of interaction with proteins have a significant impact on the distribution of drugs in the human body. In our previous study, we have shown that all analyzed pirydazinone derivatives can bind to serum albumin molecules [1]. Although albumin is the protein with the highest concentration in plasma, it is not the only one that could potentially transport and bind drugs. The study of interactions with proteins other than albumin becomes even more important in the case of pathological conditions, e.g., inflammation, in which plasma protein concentrations may differ significantly from those in a healthy organism. For this reason, we decided to analyze the interaction of the tested compounds with two additional plasma proteins: α1-acid-glycoprotein (AAG), and gamma globulins (GG). AAG is an acute-phase protein, which binds lots of pharmaceuticals, especially basic substances [11]. Its concentration increases several times in the case of inflammation, cancer, and other diseases [12]. In turn, GG belongs to proteins related to the immune system. Similarly to AAG, it can also bind metabolites, drugs, and other molecules [13,14]. Therefore, testing the interaction of new drug candidates with these two proteins seems to be justified.

Spectroscopic methods are extremely useful for studying interactions between biomacromolecules and small ligands. In this project, we combined the use of several spectroscopic methods such as UV-Vis, circular dichroism, and fluorescence with molecular modeling. This selection of research techniques allowed us to describe precisely the interactions occurring in the studied systems.

## 2. Results and Discussion

### 2.1. Interaction with ctDNA

#### 2.1.1. Molecular Docking Studies

**Figure 2:**
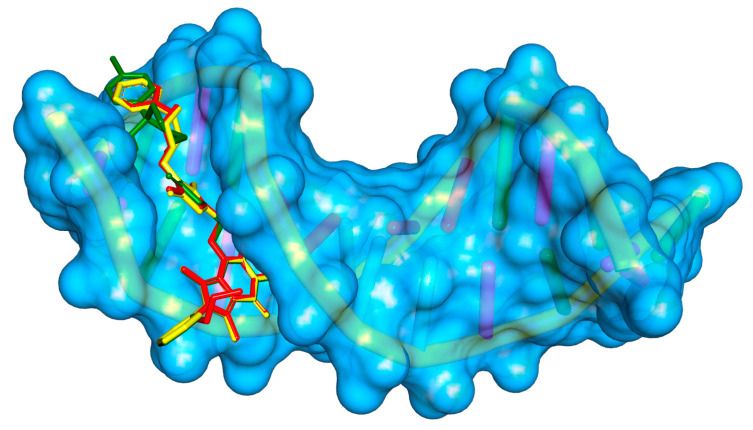
The docking position of compounds **1** (red), **3** (yellow), and **5** (green) in DNA (1vzk).

**Figure 3:**
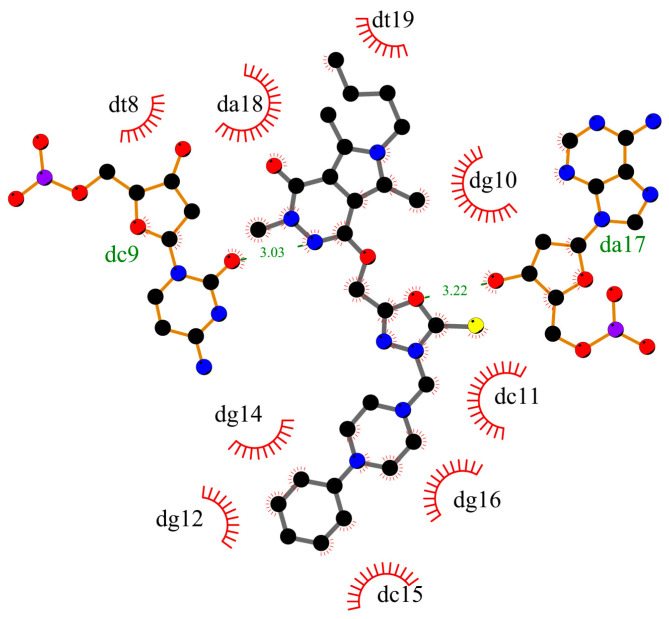
The 2D plot of interactions between molecule **1** and B-DNA.

**Figure 4:**
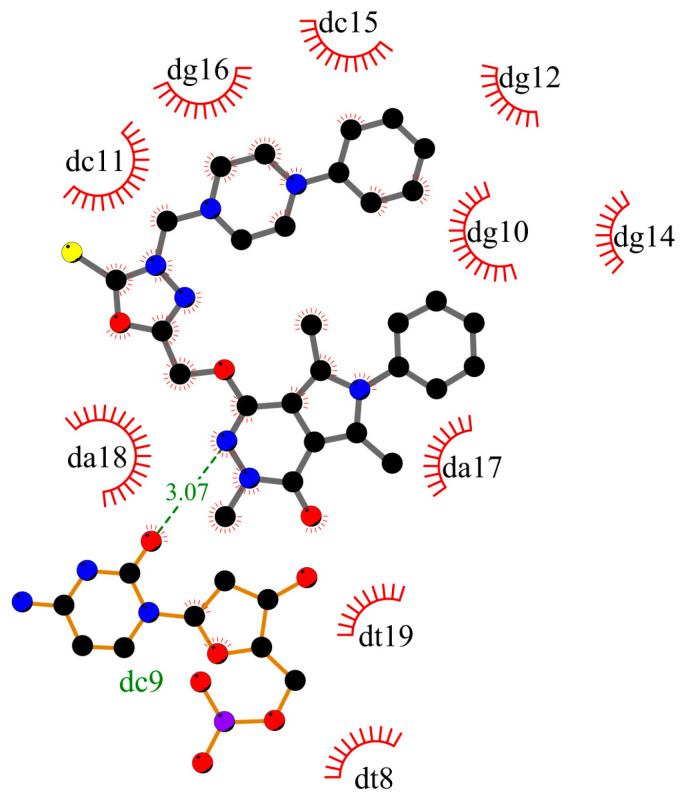
The 2D plot of interactions between molecule **3** and B-DNA.

**Figure 5:**
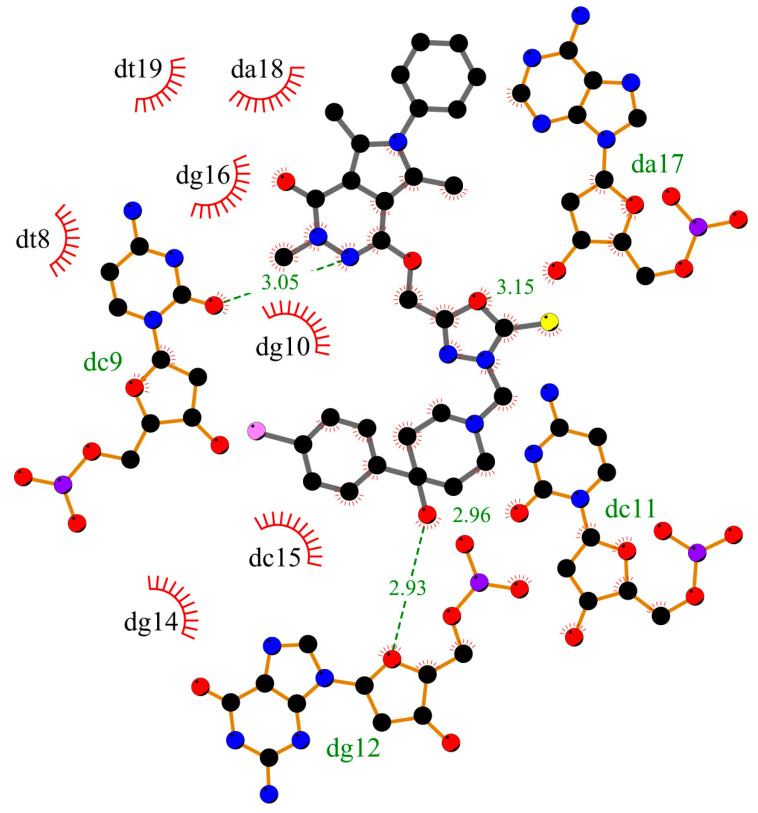
The 2D plot of interactions between molecule **5** and B-DNA.

To determine whether compounds **1**–**5** can interact with DNA, and what is the mechanism of interaction, molecular docking studies were performed. The structure of the studied compounds (due to long-chain) suggests interactions with DNA in the major or minor groove. Moreover, the compounds have planar ring moieties at the ends of the molecule which, in a preferred conformation, could be an intercalator. As a result, the mechanism of interaction with DNA would be mixed. The main part of the molecule could interact in a groove, and planar moiety in an appropriate position, after rotation, could intercalate between base pairs. The B-DNA structure was used for the simulation, PBD: ID 1vzk [15]. The grid box was set to whole DNA. Figure 2 shows the position of compound **1**, with a phenyl ring in the arylpiperazine group, compound **3**, with two phenyls, one in pyrrolo[3,4-*d*]pyridazinone moiety, second in the arylpiperazine group, and compound **5**, with substituted phenyl ring in arylpiperazine group. All compounds interact with DNA in the minor groove (compounds **2** and **4** take a similar position in the minor groove). Figure 3, Figure 4 and Figure 5 present the interaction mode, for a pose with more negative energy scoring function. Hydrogen bonds are formed: two for interaction with molecule **1**, one with molecule **3**, and four for molecule **5**. Cytosine DC9 and DC11, guanine DG12, adenine DA17, and pyrrolo[3,4-*d*]pyridazinone moiety, 2-thioxo-1,3,4-oxadiazole ring, and 4-chlorophenyl-4-hydroxypiperidyl moiety are involved in hydrogen bonds. Additionally, the complexes are stabilized by a series of hydrophobic interactions, π-alkyl, π-anion, and π-donor. The binding modes for other systems with molecules **2** and **4** are included in the Supplementary File (Figure S1a,b). It is difficult to clearly determine whether the studied molecules interact with DNA only in a minor groove or whether intercalation is also possible. This requires experimental studies. However, molecular docking has shown that all compounds interact with DNA to form stable complexes with a negative energy scoring function (Table 1). The most negative was calculated for complex with molecule 5, −10.7 kcal/mol.

#### 2.1.2. Spectroscopic Studies

##### UV-Vis Spectroscopy

**Figure 6:**
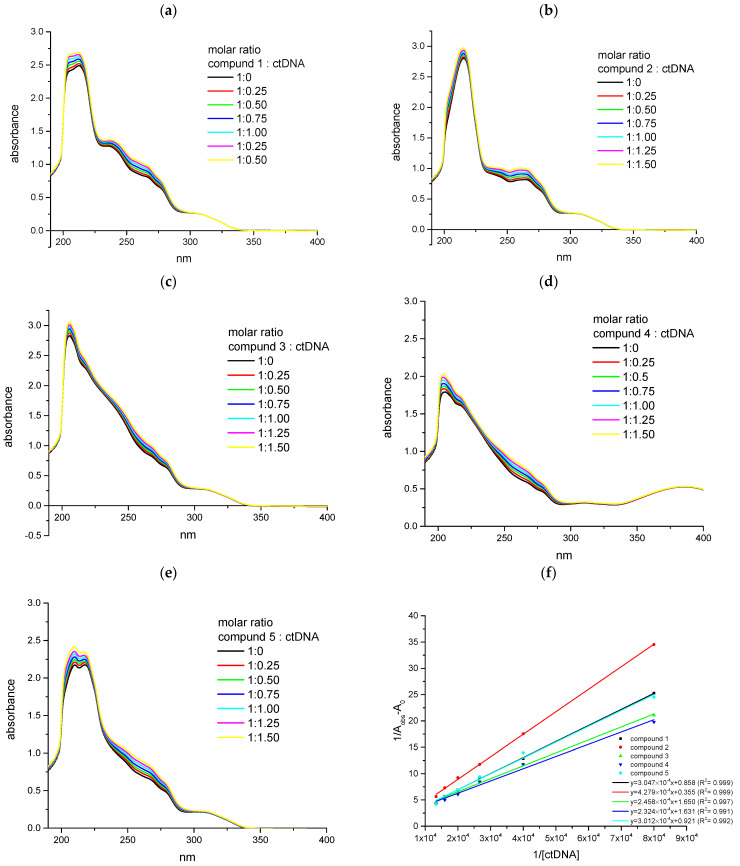
The UV-Vis spectra of complexes: (**a**) **1**, (**b**) **2**, (**c**) **3**, (**d**) **4**, and (**e**) **5** upon addition of ctDNA of varying molar ratio 0.25–1.5 with 0.25 increments (according to color legends). (**f**) The Benesi–Hildbrand plot.

The monitoring of the electronic absorption band during titration experiments shows the mechanism of interaction between compounds and ctDNA. In this study, we monitored the changes in the UV-Vis spectra of analyzed compounds with and without ctDNA solution (Figure 6). The titration of pyridazinone ligands by DNA helps to determine the mechanism by which drug derivatives and macromolecules interact with each other. In the case of strong interactions, the intensity and position of the absorption bands change. The intercalation mechanism of interaction causes hypochromic and bathochromic shifts [10,16].

In the recorded spectra for all tested compounds, a hyperchrome effect can be observed, without shifting the position of the maximum of the absorption bands (Figure 6).

In Table 2, we have collected all parameters calculated from UV-Vis spectroscopy results: the percentage of chromism, apparent association constants, and the standard of free energy Gibbs changes.

The percent of chromism effect (%H) was calculated according to Equation (1):

(1) $$\%H=\frac{A_{0}-A}{A_{0}}\cdot 100\%$$

where A_0_ is the absorption of the absence of ctDNA, and A is absorption with a maximum concentration of titration components. The apparent association constants (K_app_) were estimated by the Benesi–Hildebrand Equation (2) [17]:

(2) $$A_{obs}=\left(1-\alpha \right)c_{0}\varepsilon l+\alpha c_{0}\varepsilon_{c}l$$

where A_obs_ is the absorbance of the ctDNA solution with a different molar ratio of the compound, and ε, $\varepsilon_{c}$ are the molar absorptivity for ctDNA and the complex with the studied compound, respectively, c_0_ is the concentration of ctDNA, and l is the optical path length. The α is the association degree between interacting molecules and can be expressed as Equation (3):

(3) $$\alpha =\frac{K_{app}[compound]}{1+K_{app}[compound]}$$

According to Lambert Beer’s law (4):

A=cεl (4)

the final relationship between the absorbance changes and the K_app_ constant can be a linear function specified in the following form (5):

(5) $$\frac{1}{A_{obs}-A_{0}}=\frac{1}{A_{c}-A_{0}}+\frac{1}{K_{app}\left(A_{c}-A_{0}\right)[compound]}$$

where A_0_ is the absorbance of the ctDNA solution and A_obs_ is the absorbance of the ctDNA with a different molar ratio of the compound solution, A_c_ is the absorbance of the complex, and [compound] is the molar concentration of the compound [18].

In the next step, the free energy change was calculated, with the use of Equation (6):

(6) $$∆G^{{}^{\circ}}=-RTlnK_{app}$$

where K_app_ is the binding constant, R is the gas constant, and ΔG° is free energy change.

Obtained results suggest that analyzed pyridazinone derivatives can bind to ctDNA molecules, but intercalation is not the dominant mechanism of interaction. The chromism effect is small, and the values of K_app_, of order 10^2^–10^3^, are also too low for intercalation [19]. The highest value of the constant was obtained for compound **4**, so it can be concluded that in this case the most stable complex is formed. For example, in the case of ethidium bromide, which is a typical intercalator, the value of K_app_ is equal to 1.23 × 10^5^ [20], and it is significantly higher than those excluded in this work. It can therefore be concluded that the groove binding mechanism of interaction dominates here [21]. These results are consistent with the results obtained using molecular modeling, which indicate rather the dominance of the groove binding. However, it is necessary to assess the mechanism of interaction using other spectroscopic methods, such as fluorescence spectroscopy or CD.

##### Fluorescence Spectroscopy

*Competitive binding between ethidium bromide (EB) with ctDNA and studied compounds*

**Figure 7:**
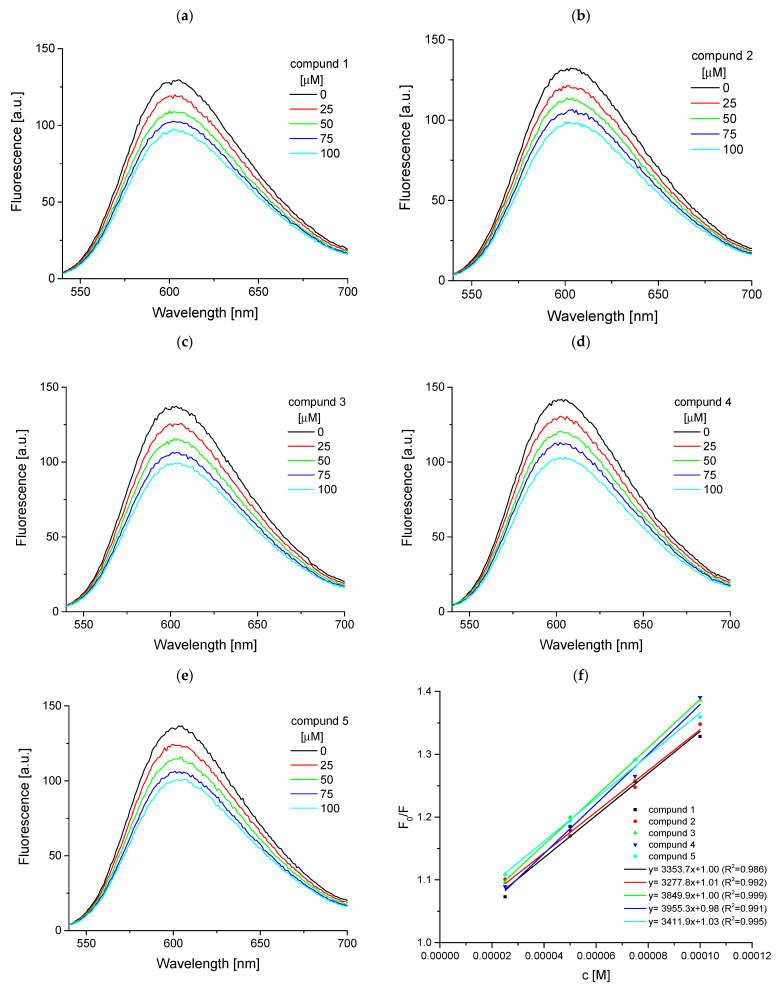
The fluorescence spectra of the competition binding between EB/ctDNA complex and compound: (**a**) **1**, (**b**) **2**, (**c**) **3**, (**d**) **4**, (**e**) **5** at 298 K, c (EB) = 10 μM, c (ctDNA) = 50 μM, c (compound) = 0–100 μM. (**f**) The plot of F_0_/F versus quencher concentration.

The competitive binding experiments of the EB/ctDNA complex with additional investigated compounds were monitored by fluorescence spectroscopy. The EB is the most widely used nucleic acid dye and it is an intercalating agent [22,23,24]. The phenanthridine rings from EB interact with stacked base pairs of double-stranded DNA by van der Walls forces and by the hydrophobic interior of the DNA molecule. The titration of EB/ctDNA complex by examined compounds was performed, and the emission spectra of absent- and present ligands were recorded. The emission signal was observed at 603 nm (Figure 7). The increasing concentrations of compounds **1**, **2**, **3**, **4**, and **5** led to a decrease in the fluorescence intensity (Figure 7). It suggests that intercalating mechanisms of interaction between studied ligands and ctDNA occur. These observations confirmed the replacement of EB in the ctDNA complex by **1**, **2**, **3**, **4**, and **5** molecules. The percentage of exchange of EB by the compound was calculated by following Equation (7):

(7) $$\%Ex=\frac{F_{0}-F}{F_{0}}\cdot 100\%$$

where F_0_ is the fluorescence intensity of complex ctDNA with EB, and F is the fluorescence intensity upon adding the studied compound. Calculated values are collected in Table 3. Over 20% EB exchange was observed in the examined concentration range, greatest for compounds **4** and **5**. By assessing the percentage of exchange, it can be concluded that it is not spectacularly large. The fluorescence results were also analyzed by the Stern–Volmer Equation (8) with inner filter correction (9):

(8) $$\frac{F_{0}}{F}=1+k_{q}\tau \left[Q\right]=1+K_{SV}$$

where F_0_ is the protein fluorescence intensity, F—protein fluorescence intensities in with the quencher, k_q_ is the quenching rate constant, τ_0_ the average fluorescence lifetime of the biomolecule, [Q] is the quencher concentration, and K_sv_ is the Stern–Volmer constant.

(9) $$F=F_{obs}10^{\frac{\left(A_{ex}+A_{em}\right)}{2}}$$

where F and F_obs_ are the corrected and observed fluorescence intensities, respectively, and A_ex_ and A_em_ are the absorbance values at excitation and emission wavelengths, respectively.

For all compounds, the Stern–Volmer constants have similar values, in the range of 3.28–3.96 × 10^3^ M^−1^ (Table 3). Therefore, it can be assumed that the mechanism of impact is rather mixed, with the predominance of other than intercalation, which is in accordance with previously described results.

*The influence of KI quenching behavior of studied compounds with present and absent ctDNA*

**Figure 8:**
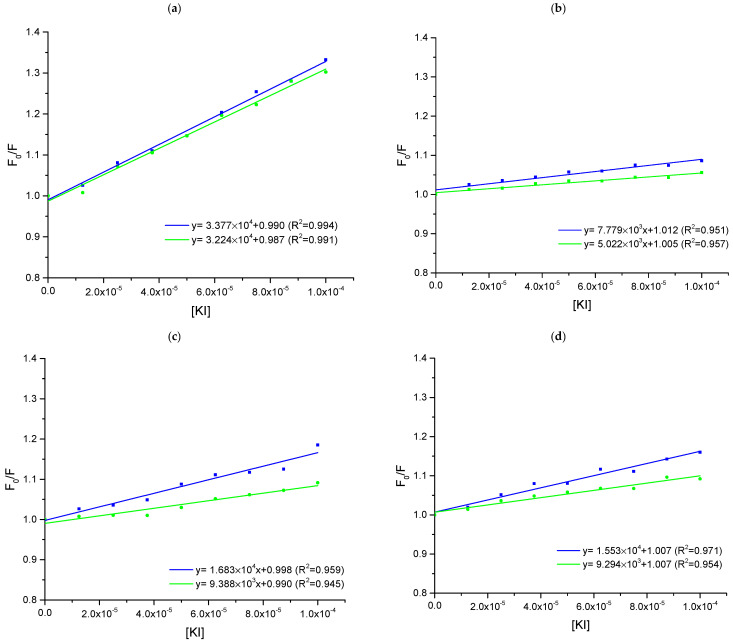
The Stern—Volmer plots for the fluorescence quenching of compounds by KI titration in with presence (green) and absence (blue) of ctDNA. The concentration of compounds and ctDNA was 50 μM and the concentration of KI was in the range 0–100 μM, the excitation wavelength (λ_ex_) were: (**a**) 240 nm—compound **1**, (**b**) 280 nm—compound **2**, (**c**) 243 nm—compound **3**, (**d**) 280 nm—compound **5**.

The next step in the study of the mechanism of interaction between ctDNA and analyzed pirydazinone derivatives was fluorescence spectroscopy with the presence of KI in the solution. In this experiment, we have measured the spectra for studied compounds and KI in the absence and presence of ctDNA (Figure 8). The comparison of Stern–Volmer constant values of both systems can suggest the type of interaction’s mechanism [25,26,27]. It was not possible to perform this experiment for compound **4** because its molecule does not have the fluorescence phenomenon. Therefore, compounds **1**, **2**, **3**, and **5** were analyzed in this way.

The highest degree of protection against the electronegative fluorophore, i.e., the I^−^ ion, is observed for intercalation, and lower for groove binding. This means that in the case of intercalation, the Stern–Volmer constant in the presence of ctDNA, should decrease significantly compared to the constant for the tested ligand only with KI. As shown in Figure 8 and Table 4, the smallest change in the magnitudes of the Stern–Volmer constants is observed for compound **1**. The percent of reduction is equal to 4.5% in this case. For the remaining three compounds, this change is slightly greater but does not exceed 45%. Therefore, based on the literature data, it can be suspected that in the case of all derivatives, groove binding or a mixed mechanism dominates [25,26,27], which is consistent with the results of previous experiments.

*The influence of the ionic strength of studied compounds with ctDNA*

The electrostatic binding mode between pyridazinone derivatives and ctDNA can be inspected by controlling the fluorescence intensity in various ionic strengths. In case of significant influence of electrostatic interaction in the binding mechanism, an increase in fluorescence intensity should be observed [28].

**Figure 9:**
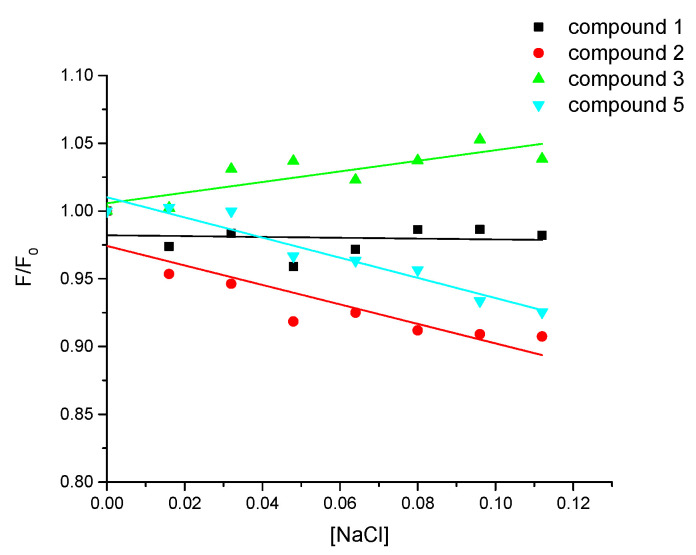
The plot of the variation in the relative fluorescence intensity (F/F_0_) of the compound/ctDNA complexes as a function of increasing concentration of NaCl from 0.112 M, compound **1**—black, compound **2**—red, compound **3**—green, compound **5**—cyan.

Compounds **2** and **5** exhibit decreased fluorescence intensity with increasing ionic strength (Figure 9). Therefore, they do not bind to the ctDNA strand electrostatically. A slight decrease was also observed for compound **1**. However, compound **3** showed an increase in fluorescence intensity with increasing NaCl concentration. It suggests the share of electrostatic binding out of the groove (Figure 9) [28].

##### Circular Dichroism Spectroscopy

**Figure 10:**
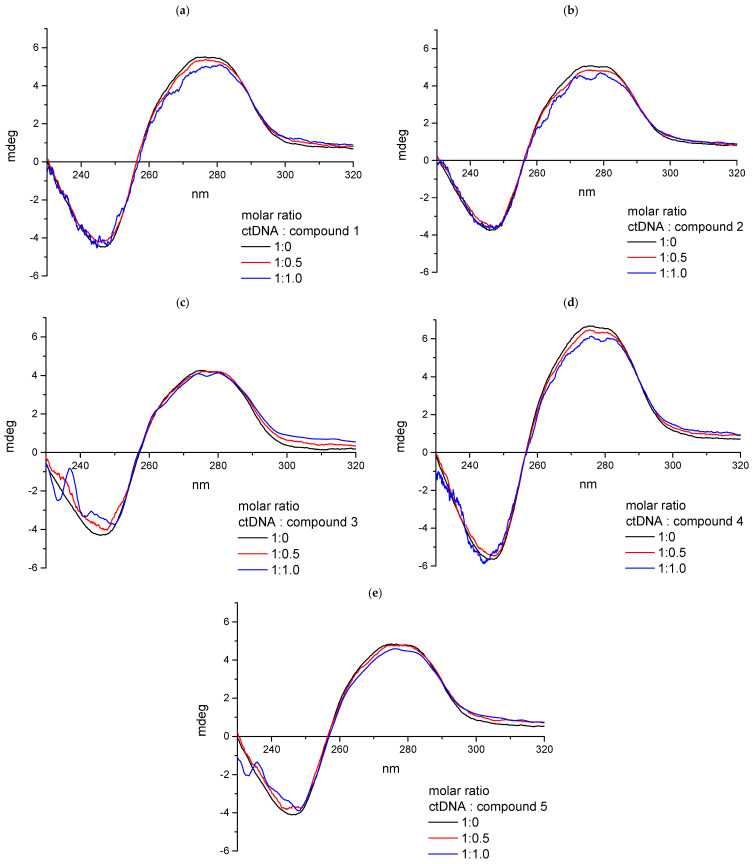
The CD spectra of ctDNA (1 × 10^−4^ mol/dm^3^) in 0.01 mol/dm^3^ phosphate buffer (pH 7.4) with the addition of varying molar ratio (0.25, 0.5, 0.75, 1.0) of: (**a**) **1**, (**b**) **2**, (**c**) **3**, (**d**) **4**, and (**e**) **5**.

Circular dichroism spectroscopy (CD) is a commonly used technique to monitor the conformation structure of peptides, proteins, or other biological fluids. The UV wavelength range gives the most valuable information. The CD spectra of the ctDNA solution can be characterized by two major peaks. The negative one at 247 nm is caused by a stacking interaction between the base pairs, and the positive peak at 278 nm is due to helicity strands (Figure 10) [29]. It is in good agreement with B conformation of DNA of double helical strand [30,31]. The DNA morphology can be changed and transformed to other forms upon interaction with small molecules, e.g., drugs. Obtained spectra are characteristic of ctDNA (Figure 10). The addition of all analyzed compounds to the ctDNA solution resulted in slightly increased noise in the CD spectra. It could be connected with distortion in the ctDNA structure which is caused by the binding reaction. However, it is worth noting that all the changes described here are small, and have a minor impact on the course of the spectra and the intensity and location of the observed bands. The very weak impact on CD signals is connected with a minor groove binding mode or an electrostatic interaction [31,32]. The presence of the intercalator has a stronger impact on the CD spectrum and causes changes within both bands [25]. It is evident that the interaction of all analyzed compounds with ctDNA does not lead to significant perturbation in the conformation of ctDNA and confirms the minor groove binding and/or an electrostatic binding manner (Figure 10). Obtained results are in agreement with UV-Vis and fluorescence study, and also with molecular modeling effects.

### 2.2. Interaction with Plasma Proteins

#### 2.2.1. Molecular Docking Studies

To determine a binding mode for interactions of studied compounds with α1-acid glycoprotein and gamma globulin inside active pocket, molecular docking simulation was made. The crystal structure from Protein Data Bank, 3kq0 (α1-acid glycoprotein) [33], and 1aj7 (gamma globulin) [34], were used. The energy scoring function for interactions is given in Table 1. For all complexes, for both AAG and GG, the energy is negative, indicating the formation of stable systems. The strongest interactions were found for molecule **5**, with a value of −10.9 kcal/mol for AAG and −9.0 for GG. For the other complexes, the energy scoring function is slightly smaller (Table 1). Moreover, systems with α1-acid glycoprotein are more stable.

**Figure 11:**
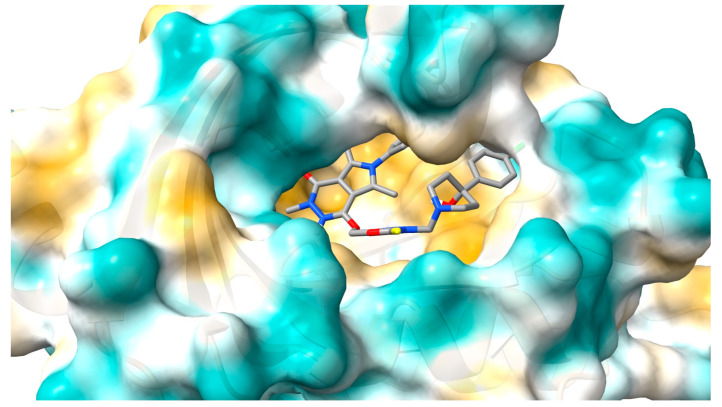
The pose of molecule **5** with the lowest energy in α1-acid glycoprotein pocket.

**Figure 12:**
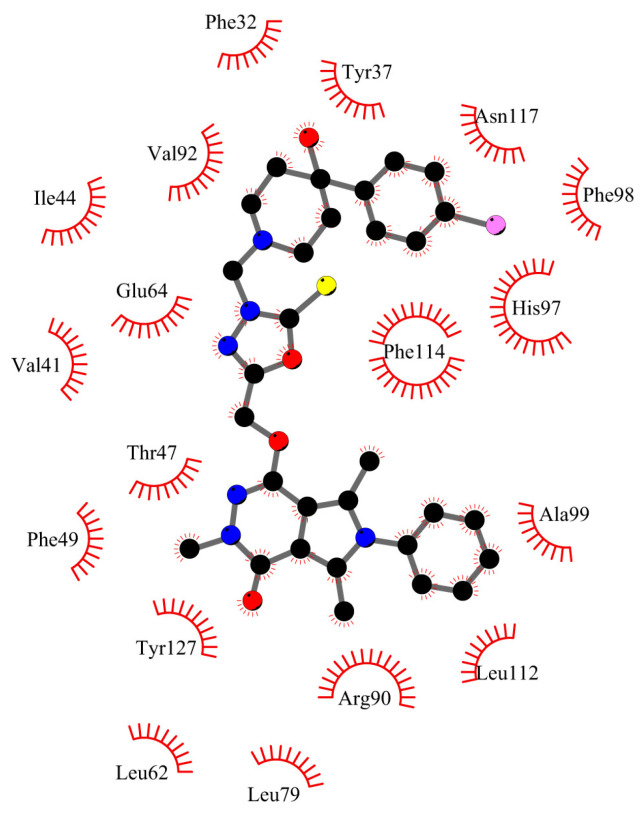
Hydrophobic interactions between compound **5** and α1-acid glycoprotein.

Figure 11 and Figure 12 show the molecule **5** orientation in the α1-acid glycoprotein pocket and a 2D plot of interactions, respectively. No hydrogen bonds were found. The system is stabilized by hydrophobic interactions. Several contact types have been found. The phenyl- pyrrolo[3,4-d]pyridazinone moiety interacts by alkyl, π-alkyl, π-π T-shaped, and π-cation contacts. 2-thioxo-1,3,4-oxadiazole ring and the chlorophenyl-4-hydroxy-1-piperidyl moiety interact by π-alkyl, alkyl, and π-donor contacts. The binding mode for other complexes is included in the Supplementary File (Figure S2a–d).

**Figure 13:**
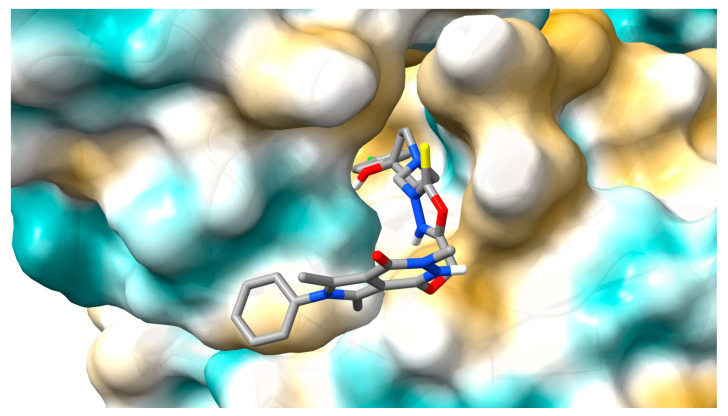
The pose of molecule **1** with the lowest energy in the gamma globulin pocket.

**Figure 14:**
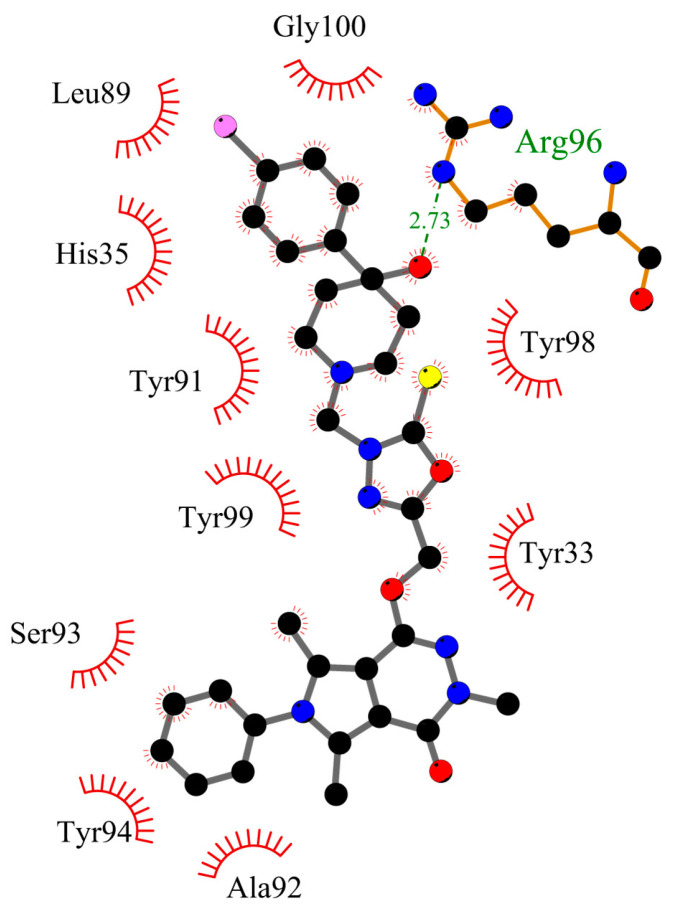
Interactions between compound **5** and the gamma globulin.

Figure 13 and Figure 14 present the compound **5** orientation in the gamma globulin pocket and 2D plot of interactions, respectively. The molecule partially goes inside the pocket. The second part, with phenyl- pyrrolo[3,4-*d*]pyridazinone moiety interacting with the GG outside. The hydrogen bond between Arg96 and -OH group from chlorophenyl-4-hydroxy-1-piperidyl moiety was found. The -Cl substituted phenyl ring interacts by hydrophobic contacts inside the pocket: π-cation with His35, π-sigma with Leu89, π-π stacked with His35, and π-alkyl with Tyr91, Tyr98, Tyr99, and Arg96. The 2-thioxo-1,3,4-oxadiazol ring via π-π T-shaped with Tyr98. Outside the pocket there are hydrophobic interactions with Tyr94 (π-π T-shaped and π-alkyl), Ala92, and Ser93 (π-alkyl). The types of interactions for other systems are included in the Supplementary File (Figure S3a–d).

#### 2.2.2. Spectroscopic Studies

##### Fluorescence Spectroscopy

Trp and Tyr are mainly responsible for the phenomenon of fluorescence in protein molecules, but the effect from the first of these amino acids is dominant. The proteins selected by us for analysis also contain tryptophanyl residues in their molecules. AAG has three Trp: Trp-25 (inside the β-barrel), Trp-122 (in the entrance to the drug-binding pocket), and Trp-166 (on the surface of the protein) [35,36], while the GG molecule contains as many as 20 Trp residues [37].

**Figure 15:**
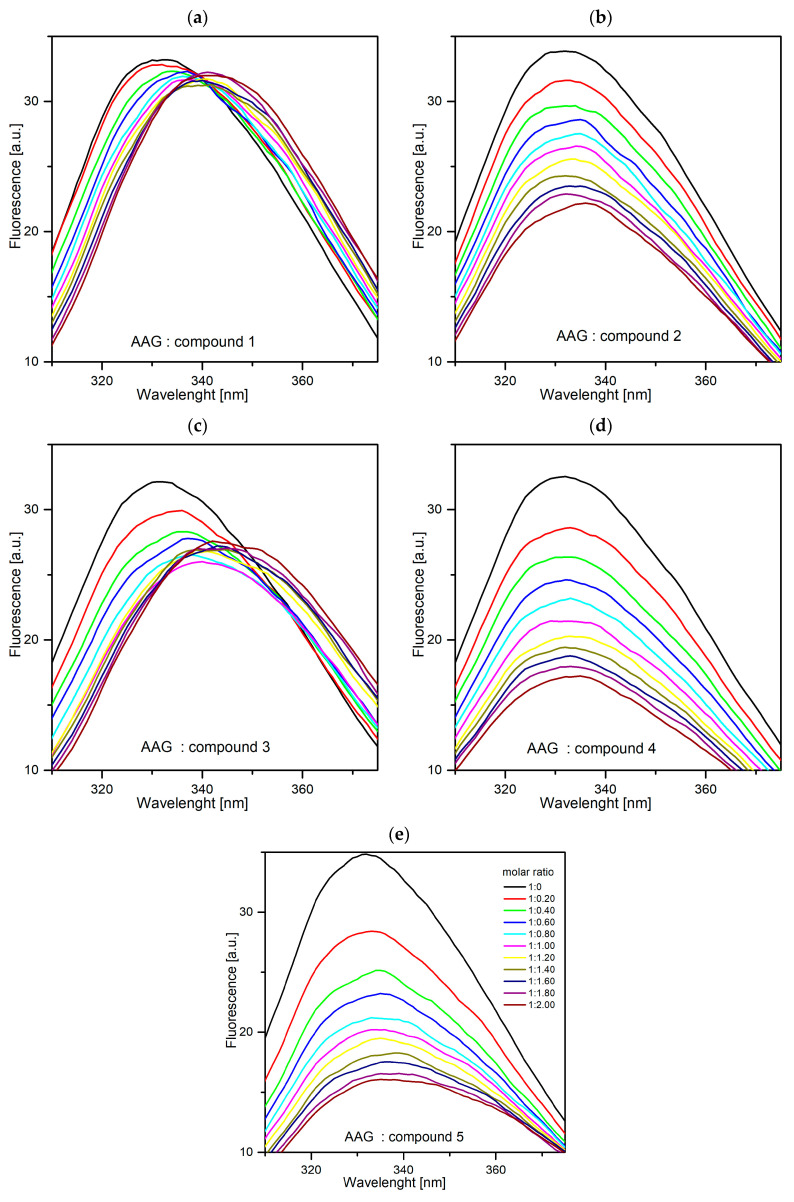
Fluorescence quenching spectra of AAG in the presence of different concentrations of compounds **1**–**5** ((**a**–**e**), respectively, T-297 K, λ_ex_ = 280 nm). The concentration of AAG was 1.0 µM, and the concentration of **1**–**5**, was: 0–2.0 µM, in the 0.2 step.

**Figure 16:**
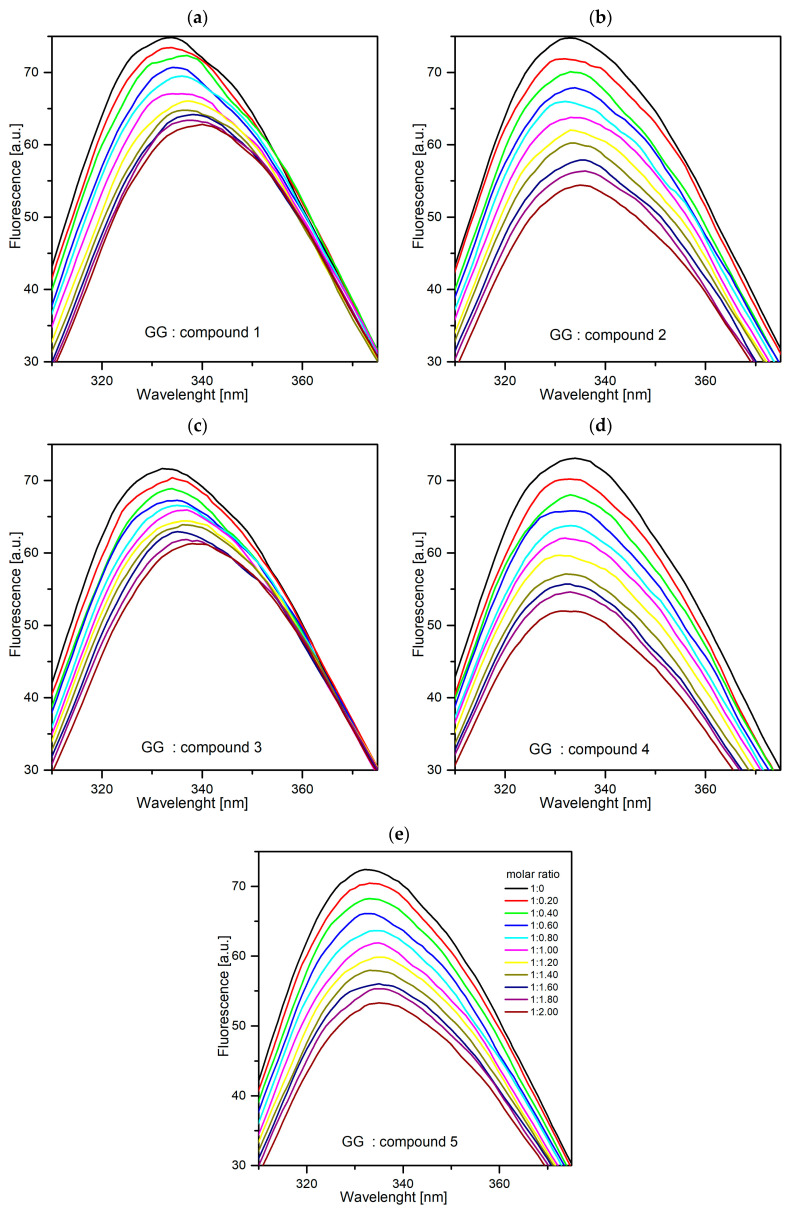
Fluorescence quenching spectra of GG in the presence of different concentrations of compounds **1**–**5** ((**a**–**e**), respectively, T-297 K, λ_ex_ = 280 nm). The concentration of GG was 1.0 µM, and the concentration of **1**–**5**, was: 0–2.0 µM, in the 0.2 step.

An experiment was carried out during which it was observed how the fluorescence intensity of the analyzed proteins changed under the influence of the increased concentration of compounds **1**–**5**. In the case of all tested systems, the fluorescence intensity decreased (Figure 15 and Figure 16). Furthermore, in the case of AAG-1, AAG-3, GG-1, and GG-3 significant hypsochromic shift of maximum emission was observed. It means that the amino acid residues are less exposed to the solvent and located in a more hydrophobic environment [38]. Based on the obtained results, it can be concluded that all pyridazinone derivatives interact with the two tested plasma proteins.

**Figure 17:**
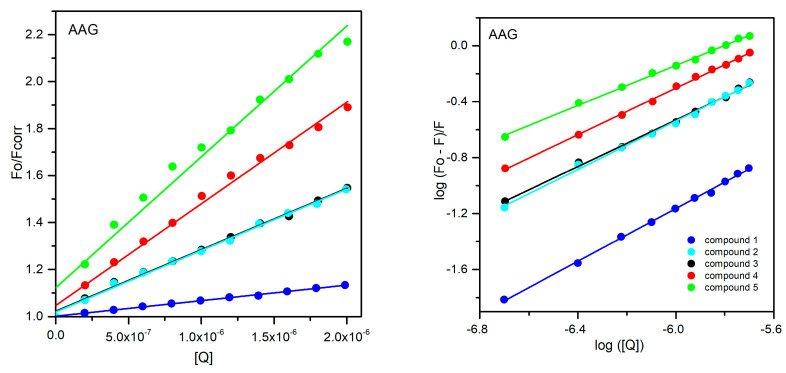
The Stern–Volmer regression plots (**left**) and double logarithm regression plots (**right**) for quenching of AAG by compounds **1**–**5**.

**Figure 18:**
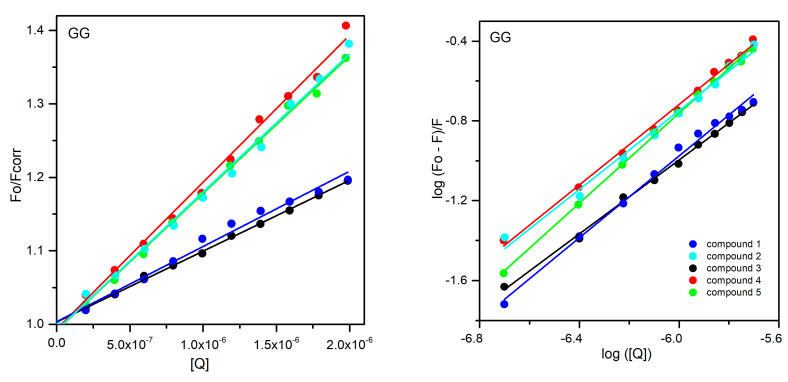
The Stern–Volmer regression plots (**left**) and double logarithm regression plots (**right**) for quenching of GG by compounds **1**–**5**.

To determine the mechanism of the above-mentioned effects, further analysis of the obtained results was carried out. Therefore, the fluorescence results were analyzed by the Stern–Volmer Equation (8) with inner filter correction (9). The Stern Volmer constants K_SV_ and the quenching rate constants k_q_ are collected in Table 5 and Table 6. The average fluorescence lifetime (τ_0_) of the biomolecule, used in the calculation, was equal to 6 ns for all proteins [39,40]. The Stern–Volmer plots are shown in Figure 17 and Figure 18 for the AAG and GG systems, respectively. The analysis of the obtained results is intended to determine whether the quenching of the fluorescence phenomenon occurs as a result of collisions of molecules in the solution (dynamic quenching) or by the formation of stable complexes between protein molecules and the analyzed compounds (static mechanism). For this purpose calculated k_q_ values can be compared with the maximum value of the quenching rate constant for the dynamic mechanism of fluorescence quenching in an aqueous solution, equal to 2 × 10^10^ L·mol^−1^·s^−1^ [41,42]. For all analyzed systems, both for AAG and GG, the obtained values are greater than the given reference value. It can therefore be concluded that all tested pyridazinone derivatives form complexes with the analyzed proteins. Moreover, analyzing the obtained results, collected in Table 5 and Table 6, it can be seen that the values of K_SV_ and k_q_ decrease with increasing temperature, which also confirms the static mechanism of fluorescence quenching.

In the next step, the fluorescence results obtained were analyzed by double logarithm regression plots, in accordance with Equation (10):

(10) $$log\frac{F_{0}-F}{F}=logK_{b}+nlog[Q]$$

The obtained curves are presented in Figure 17 and Figure 18. The use of the above formula for the analysis allows to determine the number of binding sites n in proteins for the analyzed compounds and the values of binding constants K_b_ (Table 5 and Table 6). For all tested connections between AAG and GG, and compounds **1**–**5**, the calculated values of n are values of n for all experiments approached 1, consistent with the double-logarithm model. When it comes to the values of K_b_ constants, it can be seen that for most compounds, more stable complexes are formed in systems with GG. Only for derivative **3**, this value is higher for the complex with AAG. Among the orosomucoid systems, the most stable complex is formed with compound **4**, and the least stable with compound **1**. As for complexes with GG, derivative **5** binds most strongly to this protein, and **3** the least. However, it is worth noting here that all obtained K_b_ are smaller than those obtained for interactions with serum albumin reported in our previous work [1].

The use of the fluorescence spectroscopy method also made it possible to determine the thermodynamic parameters for the ongoing processes of complex formation. Obtained values of ΔG°, ΔH°, and ΔS° are collected in Table 5 and Table 6. These parameters were calculated based on the following Equations (11) and (12):

(11) $$logK_{b}=-\frac{∆H^{{}^{\circ}}}{RT}+\frac{∆S^{{}^{\circ}}}{R}$$

(12) $$∆G^{{}^{\circ}}=∆H^{{}^{\circ}}-T∆S^{{}^{\circ}}=-RTlnK_{b}$$

where K_b_ is the binding constant, R is the gas constant, ΔH°, ΔS°, and ΔG° are enthalpy change, entropy change, and free energy change, respectively.

The analysis of the parameters described above allows to identify the type of interactions involved in the formation of complexes between proteins and the tested pyridazinone derivatives [43,44]. Negative values of ΔG° suggest the spontaneous course of reaction of the studied complexes formation. In turn, the obtained negative values of ΔH°, and ΔS° indicate the participation of hydrogen bonds and van der Waals interactions in the binding of the analyzed pyridazinone derivatives to protein molecules.

##### Circular Dichroism Spectroscopy

**Figure 19:**
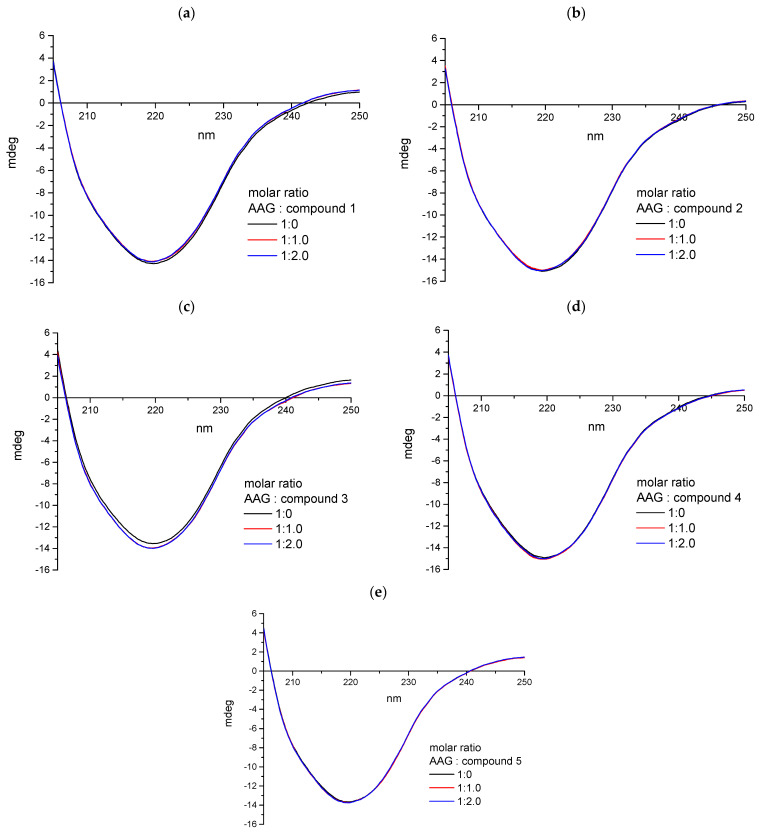
The CD spectra of AAG (1 × 10^−6^ mol/dm^3^) in 0.01 mol/dm^3^ phosphate buffer (pH 7.4) with the addition of varying molar ratio (0, 1.0, 2.0) of: (**a**) **1**, (**b**) **2**, (**c**) **3**, (**d**) **4**, (**e**) **5**.

**Figure 20:**
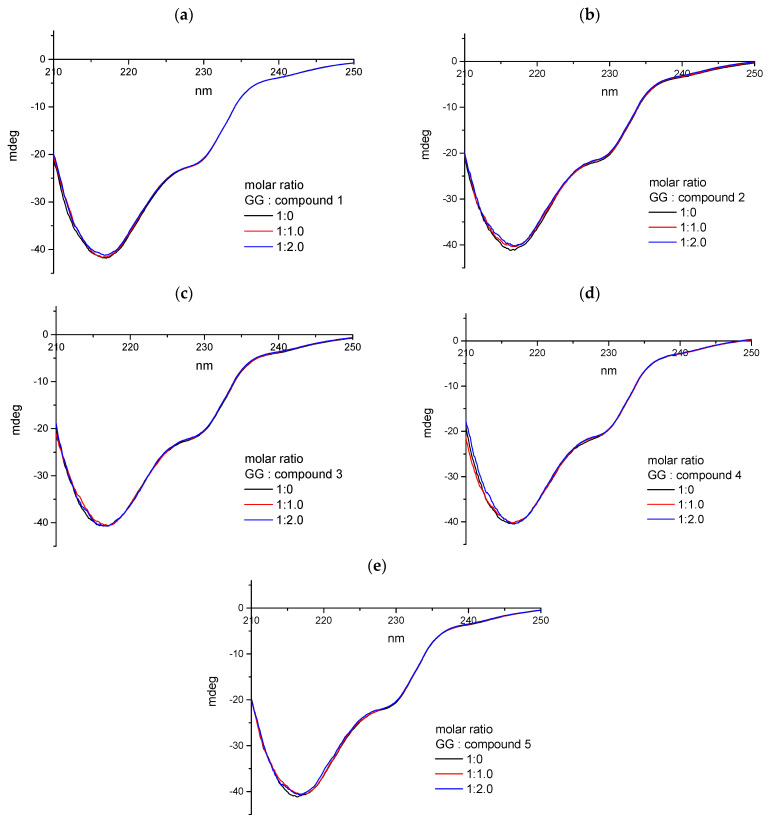
The CD spectra of GG (1 × 10^−6^ mol/dm^3^) in 0.01 mol/dm^3^ phosphate buffer (pH 7.4) with the addition of varying molar ratio (0, 1.0, 2.0) of: (**a**) **1**, (**b**) **2**, (**c**) **3**, (**d**) **4**, (**e**) **5**.

For all analyzed systems with proteins and pirydazinone derivatives, we used circular dichroism spectroscopy. Similar to the studies performed for ctDNA, the spectra measured both for solutions of proteins themselves and after adding appropriate amounts of the analyzed compounds were intended to show the influence of pyridazinone derivatives on the secondary structure of proteins. Figure 19 and Figure 20 show that spectra characteristic of both tested proteins were obtained. The analysis of the results carried out using Jasco software (Spectra Manager Version 2, CD Multivariate SSE Version 2.03.00) showed that for both AAG and GG, the dominant secondary structure is the β-sheet (Table 7 and Table 8). Both the course of the spectra and the percentage of individual secondary structures practically do not change with the increase in the concentration of the analyzed compounds in protein solutions. It can therefore be concluded that the formation of complexes between the tested plasma proteins and pyridazinone derivatives does not significantly affect the structure of AAG and GG macromolecules.

## 3. Materials and Methods

### 3.1. Materials

The synthesis of pyridazinone derivatives **1**, **2**, **3**, **4**, and **5** was described in our previous work [1]. All tested derivatives, based on their NMR and MS spectra and their physical properties, were determined to have purity of >95%. UV-Vis and fluorescence spectra of analyzed compounds are presented in Supplementary Materials (Figure S4). Studied double-stranded calf thymus DNA (ctDNA, powder), proteins: AAG and GG (powders), and 0.01 M phosphate buffer tablets were bought from Sigma-Aldrich Chemie GmbH, (St. Louis, MO, USA). Protein solutions of a given concentration were prepared by dissolving an appropriate amount of reagent in the form of powder in a phosphate buffer solution. For ctDNA, we prepared the stock solution with a concentration equal to 1 mg/mL, in phosphate buffer as a solvent. We have checked if ctDNA is free from protein. For this purpose, the absorbance in 260 and 280 nm was measured. The value of A_260_/A_280_ was higher than 1.8, which confirmed the purity of ctDNA [45]. The actual concentration of the solution was determined from Beer–Lambert law by measuring the absorbance at 260 nm and calculating it using the molar absorption coefficient equal to 6600 L/mol·cm [46]. The prepared solution was stored in a freezer, thawed, and diluted with buffer to appropriate concentrations before measurement.

### 3.2. Molecular Docking

Geometry optimization of compounds **1**–**5** was performed using DFT with a B3LYP/6-31 + G (d.p) basis set [47,48,49]. Computation was performed by the Gaussian 2016 C.01 software package [50]. The following DNA and protein structures were used: B-DNA-1vzk [15], AAG—3kq0 [33], and GG-1aj7 [34]. Ligands, DNA, and proteins were prepared using AutoDock Tools 1.5.6 [51]. Co-crystallized molecules of ligands and water were eliminated. Kollman partial charges and nonpolar hydrogens were also added. Compounds **1**–**5** were prepared by the standard procedure: rotatable bonds were ascribed, nonpolar hydrogens were merged, and partial charges were added. The molecular docking study was conducted using AutoDockVina 1.1.2 [52]. Exhaustiveness values were set as 8, 16, 24, and 60. The center of the grid box was set according to the binding pocket site in the crystal structure of the protein and to the whole DNA molecule. The docking protocol was first validated by self-docking of the crystallized ligands. The visualizations were performed using ChimeraX 1.4 software [53] and LigPlot + v.2.2 software [54].

### 3.3. Spectroscopic Studies

#### 3.3.1. Fluorescence Spectroscopy

Fluorescence spectra were measured on a Cary Eclipse 500 spectrophotometer (Agilent, Santa Clara, CA, USA).

##### Studies with Proteins

The concentrations of proteins and pirydazinone derivatives were 1.0 μM and 1.0 mM, respectively. AAG and GG solutions were prepared in phosphate buffer (pH 7.4), while the analyzed ligands were diluted in EtOH. 3 mL of a solution of each protein were used and we added a small portion of pirydazinone analogs. Experiments were performed at three temperatures: 297, 303, and 308 K. We used the following measurement parameters: 280 nm for excitation wavelength, 300–500 nm emission wavelength, and 10 mm path length. The molar ratio of compound to protein was 0.1–2.0 with 0.2 steps in all analyzed systems. All measured spectra were corrected with an inner filter during analysis. For this purpose, values were read from the UV-Vis spectra for the tested systems at the excitation and emission wavelengths. Corresponding UV-Vis spectra were measured in a quartz cuvette with a path length of 10 mm.

##### Studies with ctDNA

The emission spectra were recorded in the wavelength range of 500–700 nm and the excitation was at 525 nm. The samples were prepared in phosphate buffer (Sigma Aldrich, St. Louis, MO, USA), the final concentration of ctDNA (calf-thymus DNA, Sigma Aldrich, USA) was 50 μM, and EB (ethidium bromide, Sigma Aldrich, USA) was 20 μM. The solvents were mixed with appropriately studied compounds with a stock solutions concentration were 1 mM to achieve a molar ratio from 0.5 to 2.0 with 0.5 increments in relation to ctDNA.

The iodide potassium (KI) quenching measurements were performed for all studied pyridazinone compounds excluding compound **4**, which does not exhibit fluorescence. The exciting wavelengths were 240, 280, 243, and 280 nm for compounds **1**, **2**, **3**, and **5,** respectively. The fluorescence emission spectra were measured in the range of 300–500 nm. The final concentration of compounds and ctDNA in the mixture was 50 μM. The stock solution of KI (1 mM) was added dropwise to each mixture and then the spectra were recorded. The measurements for each compound were carried out in two layouts with and without ctDNA.

The ionic strength measurements were carried out for compounds **1**, **2**, **3**, and **5**, which are characterized by the phenomenon of fluorescence. The concentration of compounds and ctDNA was 50 μM. The NaCl (1 M) solution was added dropwise to obtain the final concentration in the mixture in the range of 0–0.112 M. Then the samples were left for 10 min to equilibrate the mixture and the emission spectra were recorded in the wavelength range 300–500 nm.

#### 3.3.2. Circular Dichroism Spectroscopy

The Jasco J-1500 magnetic circular dichroism spectrometer (JASCO International CO., Tokyo, Japan) was used for recording the CD spectra. The method of preparing the solutions of proteins and pyridazinone analogs was the same as in the fluorescence spectroscopy. The following measurement parameters were applied: the range was 205–250 nm, the scan speed rate was equal to 50 nm/min, with a response time of 1 s, path length—10 mm. The molar ratios of proteins and ligands were equal to 1:0, 1:1, and 1:2. For the analysis of the secondary structure elements we used the CD Multivariate Calibration Creation and CD Multivariate SSE programs (JASCO International CO., Tokyo, Japan). For this purpose, protein concentrations were expressed as mean residue molar concentrations.

The measurements with ctDNA were taken at wavelengths between 230 and 320 nm with 0.1 nm resolution and averaged over two scans recorded at a speed of 100 nm/min. The quartz cuvette with a path length of 10 mm was used. The ctDNA solution concentration was 100 μM in phosphate buffer (0.01 M, pH = 7.4). The sample of ctDNA was titrated by an appropriate amount of studied compounds: **1**, **2**, **3**, **4**, and **5** to achieve 0.5 and 1.0 molar ratios. The stock solutions of compounds have a 1 mM concentration. The mix samples were prepared at room temperature 297 K.

#### 3.3.3. UV-Vis Spectroscopy

The electronic spectra were recorded on UV-Vis a Jasco spectrophotometer (Jasco, Japan) over the range 190–400 nm at 0.1 nm intervals. The spectra were collected in a quartz cuvette at room temperature 297 K. The samples were prepared in phosphate buffer saline (0.01 M, pH = 7.4) and the baseline correction was made. The titration measurements were carried out increasing the ctDNA concentration with an interval of 0.25 mol and the final molar ratio was 1.5. The final ctDNA concentration was equal to 75 μM.

## 4. Conclusions

Five mono- and bi(hetero)cyclic pirydazinone derivatives, with proven anti-inflammatory and antioxidant properties, were analyzed in this study. We showed that all these molecules can form stable complexes with ctDNA, and two plasma proteins: AAG, and GG. In the case of interaction with DNA molecules, all obtained results suggest that the minor groove is a dominating mode of binding. A small contribution of the intercalation mechanism is also possible. Experimental results showed that the most stable complex is formed with compound **4**. However, for all studied pyridazinone derivatives the DNA molecule can be a molecular target. It should be emphasized that all compounds do not significantly deform the structure of the DNA molecule, so this suggests their lack of toxicity. As for the interaction with plasma proteins, more stable complexes are formed with the GG protein than with the AAG, except for compound **3**, for which the situation is the opposite. As in the case of DNA systems, compound **4** forms the strongest connections with the orosomucoid. The gamma globulin molecule interacts most strongly with derivative **5**. However, it should be emphasized that in the case of both proteins, all complexes formed are less stable than those formed with the albumin molecule. The obtained results are consistent with the previous literature reports. It has been proven that commonly used NSAIDs bind strongly to plasma proteins, especially albumin [55,56,57]. As for the bond with AAG, it is weaker for this group of compounds than in the case of albumin [58]. This does not change the fact that the interaction with AAG and GG may be important from the point of view of the pharmacodynamics and pharmacokinetics of the tested compounds.

## Figures and Tables

**Table 1:** The energy scoring function (kcal/mol) for the interaction of compounds **1**–**5** with DNA, α1-acid glycoprotein (AAG), and gamma globulin (GG).

	DNA	AAG	GG
**1**	−9.5	−9.5	−7.7
**2**	−8.8	−9.7	−7.6
**3**	−10.4	−10.6	−7.9
**4**	−10.1	−10.6	−8.8
**5**	−10.7	−10.9	−9.0

**Table 2:** The binding parameters calculated from UV-Vis spectra.

Compound	K_app_ [M^−1^]	ΔG [kJmol]	%H
**1**	2.82 × 10^3^	−1.97 × 10^4^	6.64
**2**	8.30 × 10^2^	−1.67 × 10^4^	5.97
**3**	6.71 × 10^3^	−2.18 × 10^4^	7.33
**4**	7.02 × 10^3^	−2.19 × 10^4^	11.61
**5**	3.06 × 10^3^	−1.99 × 10^4^	9.92

**Table 3:** The binding parameters K_SV_ and % of displacement for exchanging EB by studied compounds in ctDNA complexes.

Compound	K_SV_ × 10^3^ [M^−1^]	% Displacement (Ex)
**1**	3.35	24.72
**2**	3.28	25.82
**3**	3.85	27.86
**4**	3.96	28.08
**5**	3.41	29.06

**Table 4:** The K_SV_ constants for studied compounds with KI titration with present and absent of ctDNA.

Compound	K_SV_ × 10^3^ [M^−1^]		% Reduction in K_SV_
	Absent ctDNA	Present ctDNA	
**1**	33.77	32.24	4.5
**2**	7.78	5.02	35.5
**3**	16.83	9.39	44.2
**5**	15.53	9.24	40.5

**Table 5:** The Stern–Volmer constant K_sv_ and the quenching rate constant k_q_, binding constants K_b_ and number of binding sites n, thermodynamic parameters for the interaction of AAG with studied compounds at different temperatures.

		Quenching			Binding	Thermodynamic			
	T [K]	K_sv∙_10^4^ [dm^3^·mol^−1^]	k_q∙_10^12^ [dm^3^·mol^−1^·s^−1^]	logK_b_	K_b∙_10^4^ [dm^3^·mol^−1^]	n	ΔG° [kJ∙mol^−1^]	ΔH° [kJ∙mol^−1^]	ΔS° [J∙mol^−1^∙K^−1^]
1	297	6.60 ± 0.10	11.0	4.49 ± 0.07	3.09	0.94 ± 0.01	−27.11	−155.65	−432.83
	303	4.94 ± 0.18	8.23	4.10 ± 0.30	1.26	0.90 ± 0.05			
	308	3.55 ± 0.27	5.92	3.49 ± 0.29	0.31	0.82 ± 0.08			
2	297	26.15 ± 0.53	43.58	4.66 ± 0.10	4.57	0.87 ± 0.02	−26.17	−109.55	−280.76
	303	13.58 ± 0.59	22.63	4.17 ± 0.15	1.48	0.83 ± 0.03			
	308	13.45 ± 0.68	22.42	3.98 ± 0.20	0.95	0.84 ± 0.02			
3	297	26.12 ± 0.56	43.53	4.66 ± 0.10	4.57	0.87 ± 0.02	−25.40	−159.25	−450.70
	303	13.68 ± 0.57	22.80	3.91 ± 0.26	0.81	0.79 ± 0.04			
	308	12.30 ± 0.86	20.50	3.47 ± 0.30	0.29	0.71 ± 0.08			
4	297	43.26 ± 1.24	72.10	4.70 ± 0.07	5.01	0.83 ± 0.02	−26.78	−80.00	−165.70
	303	23.88 ± 1.40	39.80	4.47 ± 0.23	2.95	0.84 ± 0.04			
	308	20.21 ± 1.37	33.68	4.22 ± 0.18	1.66	0.81 ± 0.03			
5	297	55.71± 2.78	92.85	4.15 ± 0.07	1.41	0.72 ± 0.02	−23.77	−93.87	−236.03
	303	23.91 ± 1.43	39.85	3.95 ± 0.13	0.89	0.76 ± 0.02			
	308	22.56 ± 1.82	37.6	3.54 ± 0.12	0.35	0.69 ± 0.02			

**Table 6:** The Stern–Volmer constant K_sv_ and the quenching rate constant k_q_, binding constants K_b_ and number of binding sites n, thermodynamic parameters for the interaction of GG with studied compounds at different temperatures.

		Quenching			Binding	Thermodynamic			
	T [K]	K_sv∙_10^4^ [dm^3^·mol^−1^]	k_q∙_10^12^ [dm^3^·mol^−1^·s^−1^]	logK_b_	K_b∙_10^4^ [dm^3^·mol^−1^]	n	ΔG° [kJ∙mol^−1^]	ΔH° [kJ∙mol^−1^]	ΔS° [J∙mol^−1^∙K^−1^]
1	297	10.24 ± 0.36	17.07	5.19 ± 0.17	15.48	1.03 ± 0.03	−29.44	−155.02	−422.85
	303	9.29 ± 0.33	15.48	4.60 ± 0.14	3.98	0.94 ± 0.04			
	308	7.14 ± 0.22	11.90	4.22 ± 0.15	1.66	0.89 ± 0.03			
2	297	18.81 ± 0.49	31.35	5.19 ± 0.20	15.49	0.99 ± 0.04	−29.67	−97.62	−228.80
	303	11.15 ± 0.39	18.58	4.94 ± 0.24	8.71	0.98 ± 0.03			
	308	7.21 ± 0.20	12.02	4.57 ± 0.23	3.72	0.95 ± 0.04			
3	297	9.65 ± 0.11	16.08	4.57 ± 0.09	3.72	0.93 ± 0.02	−26.13	−78.71	−177.09
	303	9.20 ± 0.41	15.33	4.37 ± 0.10	2.34	0.90 ± 0.03			
	308	7.11 ± 0.34	11.85	4.07 ± 0.13	1.17	0.86 ± 0.04			
4	297	20.01 ± 0.48	33.35	5.34 ± 0.13	21.88	1.01 ± 0.02	−30.32	−71.87	−139.92
	303	16.00 ± 0.46	26.67	5.07 ± 0.21	11.75	0.98 ± 0.04			
	308	14.67 ± 0.55	24.45	4.89 ± 0.17	7.76	0.95 ± 0.03			
5	297	18.68± 0.33	31.13	6.01 ± 0.08	102.33	1.12 ± 0.02	−34.12	−54.02	−67.01
	303	16.05 ± 0.44	26.75	5.83 ± 0.20	67.61	1.11 ± 0.04			
	308	12.94 ± 0.74	21.57	5.66 ± 0.23	45.71	1.10 ± 0.04			

**Table 7:** The percentage of content of the secondary structure elements in AAG in the absence and presence of analyzed pyridazinone derivatives **1**–**5** and Root Mean Square Deviation (RMSD) for all analyzed systems. The RMSD was determined based on the experimental spectra and those calculated in CD Multivariate SSE program.

AAG: Analyzed Compound Molar Ratio	% α-Helix	% β-Sheet	% β-Turn	% Other	RMSD
Compound **1**					
1:0	21.5	35.9	10.7	31.8	0.0025
1:1	21.0	36.1	10.7	32.2	0.0022
1:2	21.0	36.1	10.7	32.1	0.0023
Compound **2**					
1:0	22.2	35.6	10.9	31.3	0.0024
1:1	22.0	35.8	10.9	31.3	0.0025
1:2	22.1	35.7	10.9	31.3	0.0024
Compound **3**					
1:0	21.3	36.6	10.6	31.6	0.0024
1:1	21.2	36.3	10.6	31.9	0.0024
1:2	21.2	36.3	10.6	32.0	0.0025
Compound **4**					
1:0	21.6	35.9	10.8	31.6	0.0024
1:1	21.9	35.9	10.8	31.4	0.0026
1:2	21.8	36.0	10.8	31.3	0.0023
Compound **5**					
1:0	21.2	36.4	10.5	31.8	0.0020
1:1	21.4	36.3	10.5	31.8	0.0023
1:2	21.3	36.5	10.5	31.7	0.0024

**Table 8:** The percentage of content of the secondary structure elements in GG in the absence and presence of analyzed pyridazinone derivatives **1**–**5** and Root Mean Square Deviation (RMSD) for all analyzed systems. The RMSD was determined based on the experimental spectra and those calculated in CD Multivariate SSE program.

GG: Analyzed Compound Molar Ratio	% α-Helix	% Β-Sheet	% β-Turn	% Other	RMSD
Compound **1**					
1:0	9.4%	38.0%	13.3%	39.3%	0.0021
1:1	9.3%	38.2%	13.3%	39.2%	0.0020
1:2	9.3%	38.1%	13.3%	39.2%	0.0023
Compound **2**					
1:0	9.1%	38.1%	13.3%	39.4%	0.0020
1:1	8.9%	38.0%	13.4%	39.7%	0.0022
1:2	8.8%	38.1%	13.4%	39.7%	0.0018
Compound **3**					
1:0	9.2%	38.0%	13.4%	39.4%	0.0023
1:1	9.1%	38.0%	13.4%	39.5%	0.0022
1:2	9.1%	38.2%	13.3%	39.3%	0.0022
Compound **4**					
1:0	8.9%	38.1%	13.3%	39.6%	0.0022
1:1	8.8%	37.9%	13.4%	40.0%	0.0020
1:2	8.8%	38.4%	13.3%	39.5%	0.0018
Compound **5**					
1:0	9.2%	38.1%	13.3%	39.3%	0.0022
1:1	9.0%	38.0%	13.4%	39.5%	0.0023
1:2	9.0%	38.1%	13.4%	39.5%	0.0021

## Data Availability

Data is contained within the Supplementary Material.

## Supplementary Materials

The following supporting information can be downloaded at: https://www.mdpi.com/article/10.3390/ijms25031784/s1.

## References

[1] Szczukowski Ł., Redzicka A., Wiatrak B., Krzyżak E., Marciniak A., Gębczak K., Gębarowski T., Świątek P. (2020). Design, synthesis, biological evaluation and in silico studies of novel pyrrolo[3,4-d]pyridazinone derivatives with promising anti-inflammatory and antioxidant activity. Bioorg. Chem..

[2] Asif M. (2017). Various Chemical and Biological Activities of Pyridazinone Derivatives. Cent. Eur. J. Exp. Biol..

[3] Alam M.T., Asif M. (2019). Pharmacological activities of pyridazines and pyridazinone Derivatives: A Review on biologically active scaffold. South Asian Res. J. Pharm. Sci..

[4] Imran M. (2020). Mohammad Asif Biologically Active Pyridazines and Pyridazinone Derivatives: A Scaffold for the Highly Functionalized Compounds. Russ. J. Bioorganic Chem..

[5] Akhtar W., Shaquiquzzaman M., Akhter M., Verma G., Khan M.F., Alam M.M. (2016). The therapeutic journey of pyridazinone. Eur. J. Med. Chem..

[6] Szandruk-Bender M., Merwid-Ląd A., Wiatrak B., Danielewski M., Dzimira S., Szkudlarek D., Szczukowski Ł., Świątek P., Szeląg A. (2021). Novel 1,3,4-oxadiazole derivatives of pyrrolo [3,4-d]pyridazinone exert anti-inflammatory activity without acute gastrotoxicity in the carrageenan-induced rat paw edema test. J. Inflamm. Res..

[7] Szandruk-Bender M., Wiatrak B., Dzimira S., Merwid-Ląd A., Szczukowski Ł., Świątek P., Szeląg A. (2022). Targeting Lineage-Specific Transcription Factors and Cytokines of the Th17/Treg Axis by Novel 1,3,4-Oxadiazole Derivatives of Pyrrolo[3,4-d]pyridazinone Attenuates TNBS-Induced Experimental Colitis. Int. J. Mol. Sci..

[8] Szandruk-Bender M., Wiatrak B., Szczukowski Ł., Świątek P., Rutkowska M., Dzimira S., Merwid-Ląd A., Danielewski M., Szeląg A. (2020). Novel 1,3,4-oxadiazole derivatives of pyrrolo[3,4-d]pyridazinone exert antinociceptive activity in the tail-flick and formalin test in rodents and reveal reduced gastrotoxicity. Int. J. Mol. Sci..

[9] Nunn C.M., Van Meervelt L., Zhang S., Moore M.H., Kennard O. (1991). DNA-drug interactions. The crystal structures of d(TGTACA) and d(TGATCA) complexed with daunomycin. J. Mol. Biol..

[10] Sirajuddin M., Ali S., Badshah A. (2013). Drug-DNA interactions and their study by UV-Visible, fluorescence spectroscopies and cyclic voltametry. J. Photochem. Photobiol. B Biol..

[11] Fitos I., Visy J., Zsila F., Bikádi Z., Mády G., Simonyi M. (2004). Specific ligand binding on genetic variants of human α 1-acid glycoprotein studied by circular dichroism spectroscopy. Biochem. Pharmacol..

[12] Zsila F., Molnár P., Deli J., Lockwood S.F. (2005). Circular dichroism and absorption spectroscopic data reveal binding of the natural cis-carotenoid bixin to human α1-acid glycoprotein. Bioorg. Chem..

[13] Ying L., Chao W., Guanghua L. (2010). Interaction of Jatrorrhizine with human gamma globulin in membrane mimetic environments: Probing of the binding mechanism and binding site by spectroscopic and molecular modeling methods. J. Mol. Struct..

[14] Li X., Wang X., Liu H., Peng Y., Yan Y., Ni T. (2021). Mechanism evaluation of the interactions between eight flavonoids and γ-globulin based on multi-spectroscopy. J. Mol. Struct..

[15] Mallena S., Lee M.P.H., Bailly C., Neidle S., Kumar A., Boykin D.W., Wilson W.D. (2004). Thiophene-based diamidine forms a “super” AT binding minor groove agent. J. Am. Chem. Soc..

[16] Hasi Q.M., Guo Y., Yang J., Mu X., Chen L., Wang S., Xiao C., Zhang Y., Han Z. (2022). Synthesis, DNA-binding abilities, and in vitro antitumor activity of water-soluble copper porphyrin and its Schiff-base complexes. New J. Chem..

[17] Benesi H.A., Hildebrand J.H. (1949). A Spectrophotometric Investigation of the Interaction of Iodine with Aromatic Hydrocarbons. J. Am. Chem. Soc..

[18] Salem A.A., Lotfy M., Amin A., Ghattas M.A. (2019). Characterization of human serum albumin’s interactions with safranal and crocin using multi-spectroscopic and molecular docking techniques. Biochem. Biophys. Rep..

[19] Sharfalddin A.A., Emwas A.H., Jaremko M., Hussien M.A. (2021). Synthesis and theoretical calculations of metal-antibiotic chelation with thiamphenicol:in vitroDNA and HSA binding, molecular docking, and cytotoxicity studies. New J. Chem..

[20] Popović A., Nikolić M., Mijajlović M., Ratković Z., Jevtić V., Trifunović S.R., Radić G., Zarić M., Canović P., Milovanović M. (2019). DNA binding and antitumor activities of zinc(II) complexes with some S-alkenyl derivatives of thiosalicylic acid. Transit. Met. Chem..

[21] Arif R., Nayab P.S., Ansari I.A., Shahid M., Irfan M., Alam S., Abid M. (2018). Rahisuddin Synthesis, molecular docking and DNA binding studies of phthalimide-based copper(II) complex: In vitro antibacterial, hemolytic and antioxidant assessment. J. Mol. Struct..

[22] Vardevanyan P.O., Antonyan A.P., Parsadanyan M.A., Davtyan H.G., Karapetyan A.T. (2003). The binding of ethidium bromide with DNA: Interaction with single- and double-stranded structures. Exp. Mol. Med..

[23] Olmsted J., Kearns D.R. (1977). Mechanism of Ethidium Bromide Fluorescence Enhancement on Binding to Nucleic Acids. Biochemistry.

[24] Sigmon J., Larcom L.L. (1996). The effect of ethidium bromide on mobility of DNA fragments in agarose gel electrophoresis. Electrophoresis.

[25] Nayab P.S., Pulaganti M., Chitta S.K., Oves M. (2015). Rahisuddin Synthesis, spectroscopic studies of novel N-substituted phthalimides and evaluation of their antibacterial, antioxidant, DNA binding and molecular docking studies. Bangladesh J. Pharmacol..

[26] Zhang G., Hu X., Fu P. (2012). Spectroscopic studies on the interaction between carbaryl and calf thymus DNA with the use of ethidium bromide as a fluorescence probe. J. Photochem. Photobiol. B Biol..

[27] Jana B., Senapati S., Ghosh D., Bose D., Chattopadhyay N. (2012). Spectroscopic exploration of mode of binding of ctDNA with 3-hydroxyflavone: A contrast to the mode of binding with flavonoids having additional hydroxyl groups. J. Phys. Chem. B.

[28] Wani T.A., Alsaif N., Bakheit A.H., Zargar S., Al-Mehizia A.A., Khan A.A. (2020). Interaction of an abiraterone with calf thymus DNA: Investigation with spectroscopic technique and modelling studies. Bioorg. Chem..

[29] Uma V., Kanthimathi M., Weyhermuller T., Nair B.U. (2005). Oxidative DNA cleavage mediated by a new copper (II) terpyridine complex: Crystal structure and DNA binding studies. J. Inorg. Biochem..

[30] Kypr J., Kejnovská I., Renciuk D., Vorlícková M. (2009). Circular dichroism and conformational polymorphism of DNA. Nucleic Acids Res..

[31] Arjmand F., Jamsheera A., Mohapatra D.K. (2013). Synthesis, characterization and in vitro DNA binding and cleavage studies of Cu(II)/Zn(II) dipeptide complexes. J. Photochem. Photobiol. B Biol..

[32] Karidi K., Garoufis A., Tsipis A., Hadjiliadis N., Den Dulk H., Reedijk J. (2005). Synthesis, characterization, in vitro antitumor activity, DNA-binding properties and electronic structure (DFT) of the new complex cis-(Cl,Cl)[RuIICl2(NO+)(terpy)]Cl. Dalt. Trans..

[33] Schönfeld D.L., Ravelli R.B.G., Mueller U., Skerra A. (2008). The 1.8-Å Crystal Structure of α1-Acid Glycoprotein (Orosomucoid) Solved by UV RIP Reveals the Broad Drug-Binding Activity of This Human Plasma Lipocalin. J. Mol. Biol..

[34] Wedemayer G.J., Patten P.A., Wang L.H., Schultz P.G., Stevens R.C. (1997). Structural insights into the evolution of an antibody combining site. Science.

[35] Azad M.A.K., Huang J.X., Cooper M.A., Roberts K.D., Thompson P.E., Nation R.L., Li J., Velkov T. (2012). Structure–activity relationships for the binding of polymyxins with human α-1-acid glycoprotein. Biochem. Pharmacol..

[36] Albani J.R. (2003). Relation between the secondary structure of carbohydrate residues of α1-acid glycoprotein (orosomucoid) and the fluorescence of the protein. Carbohydr. Res..

[37] Owczarzy A., Zięba A., Pożycka J., Kulig K., Rogóż W., Szkudlarek A., Maciążek-jurczyk M. (2021). Spectroscopic studies of quinobenzothiazine derivative in terms of the in vitro interaction with selected human plasma proteins. Part 1. Molecules.

[38] Chen G.Z., Huang X.Z., Xu J.G., Zneng Z.Z., Wang Z.B. (1990). The Methods of Fluorescence Analysis.

[39] Phys J.C., Lakowicz J.R., Weber G. (1972). Quenching of Fluorescence by Oxygen. A Probe for Structural Fluctuations in Macromoleculest. Biochemistry.

[40] Anand U., Mukherjee S. (2013). Reversibility in protein folding: Effect of β-cyclodextrin on bovine serum albumin unfolded by sodium dodecyl sulphate. Phys. Chem. Chem. Phys..

[41] Ware W.R. (1962). Oxygen quenching of fluorescence in solution: An experimental study of the diffusion process. J. Phys. Chem..

[42] Lakowicz J.R. (2006). Principles of Fluorescence Spectroscopy.

[43] Klotz I.M., Urquhart J.M. (1949). The Binding of Organic Ions by Proteins. Effect of Temperature. J. Am. Chem. Soc..

[44] Ross P.D., Subramanian S. (1981). Thermodynamics of Protein Association Reactions: Forces Contributing to Stability. Biochemistry.

[45] Shahabadi N., Mohammadi S., Alizadeh R. (2011). DNA interaction studies of a new platinum(II) complex containing different aromatic dinitrogen ligands. Bioinorg. Chem. Appl..

[46] Marmur J. (1961). A procedure for the isolation of deoxyribonucleic acid from micro-organisms. J. Mol. Biol..

[47] Becke A.D. (1993). Density-functional thermochemistry. III. The role of exact exchange. J. Chem. Phys..

[48] Lee C., Yang W., Parr R.G. (1988). Development of the Colle-Salvetti correlation-energy formula into a functional of the electron density. Phys. Rev. B.

[49] Perdew J.P., Wang Y. (1992). Accurate and simple analytic representation of the electron-gas correlation energy. Phys. Rev. B.

[50] Frisch M.J., Trucks G.W., Schlegel H.B., Scuseria G.E., Robb M.A., Cheeseman J.R., Scalmani G., Barone V., Petersson G.A., Nakatsuji H. Gaussian~16 {R}evision {C}.01 2016. https://gaussian.com/citation/.

[51] Morris G.M., Huey R., Lindstrom W., Sanner M.F., Belew R.K., Goodsell D.S., Olson A.J. (2009). AutoDock4 and AutoDockTools4: Automated Docking with Selective Receptor Flexibility. J. Comput. Chem..

[52] Trott O., Olson A.J. (2010). AutoDock Vina: Improving the speed and accuracy of docking with a new scoring function, efficient optimization, and multithreading. J. Comput. Chem..

[53] Pettersen E.F., Goddard T.D., Huang C.C., Meng E.C., Couch G.S., Croll T.I., Morris J.H., Ferrin T.E. (2021). UCSF ChimeraX: Structure visualization for researchers, educators, and developers. Protein Sci..

[54] Laskowski R.A., Swindells M.B. (2011). LigPlot+: Multiple LigandÀProtein Interaction Diagrams for Drug Discovery. J. Chem. Inf. Model.

[55] Wanwimolruk S., Brooks P., Birkett D. (1983). Protein binding of non-steroidal anti-inflammatory drugs in plasma and synovial fluid of arthritic patients. Br. J. Clin. Pharmacol..

[56] Lin J.H., Cocchetto D.M., Duggan D.E. (1987). Protein Binding as a Primary Determinant of the Clinical Pharmacokinetic Properties of Non-Steroidal Anti-Inflammatory Drugs. Clin. Pharmacokinet..

[57] Montero M.T., Estelrich J., Valls O. (1990). Binding of non-steroidal anti-inflammatory drugs to human serum albumin. Int. J. Pharm..

[58] Israili Z.H., Dayton P.G. (2001). Human alpha-1-glycoprotein and its interactions with drugs. Drug Metab. Rev..

