# Supplementary material for: Anti-Inflammatory and Antioxidant Pyrrolo[3,4-d]pyridazinone Derivatives Interact with DNA and Bind to Plasma Proteins—Spectroscopic and In Silico Studies
Source: Int J Mol Sci. 2024 Feb 1;25(3):1784. doi: 10.3390/ijms25031784 (PMC10855066; doi:10.3390/ijms25031784)
Supplement: Supplementary file 1 [file ijms-25-01784-s001.zip › ijms-2851439-supplementary.pdf]

Supplementary materials

Anti-inflammatory and antioxidant pyrrolo[3,4-d]pyridazinone derivatives interact with DNA and bind to plasma proteins – spectroscopic and In Silico studies

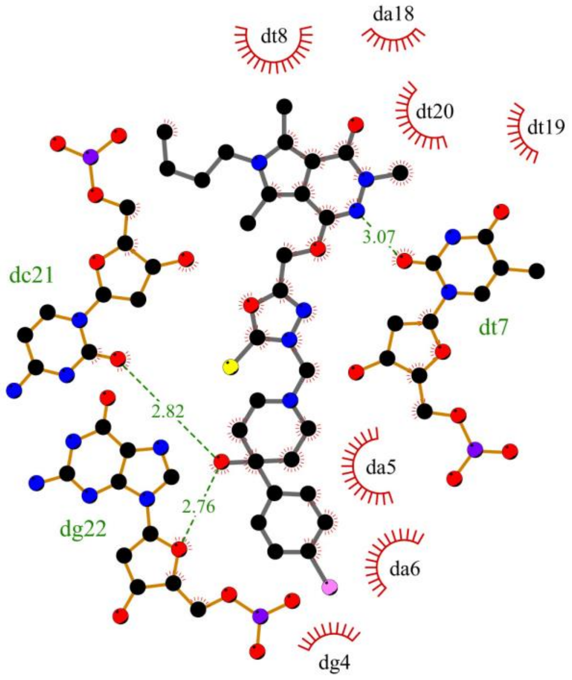

Figure S1a. The 2D plot of interactions between molecule 2 and B-DNA.

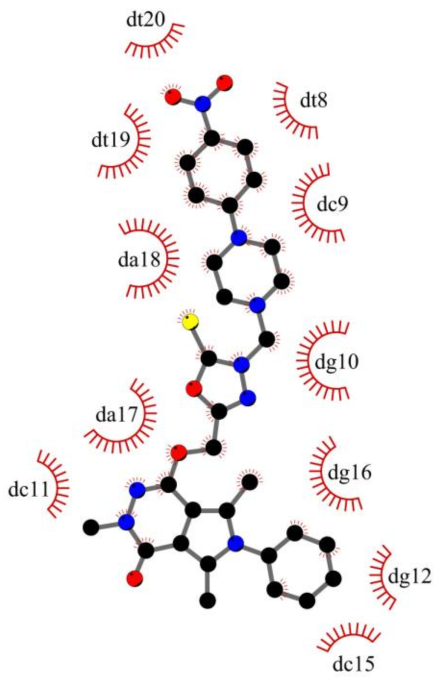

Figure S1b. The 2D plot of interactions between molecule 4 and B-DNA.

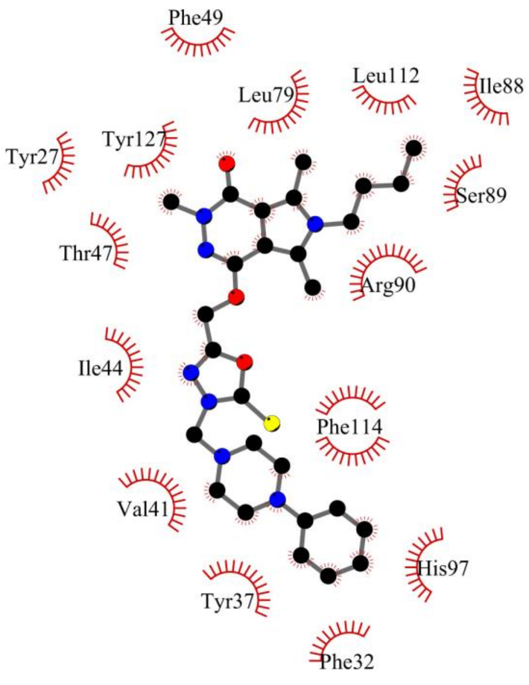

Figure S2a. Hydrophobic interactions between compound 1 and α1-acid glycoprotein.

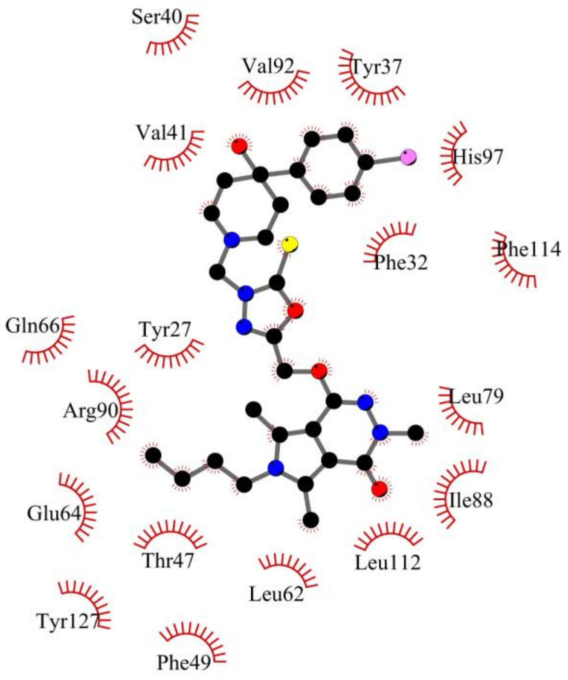

Figure S2b. Hydrophobic interactions between compound 2 and α1-acid glycoprotein.

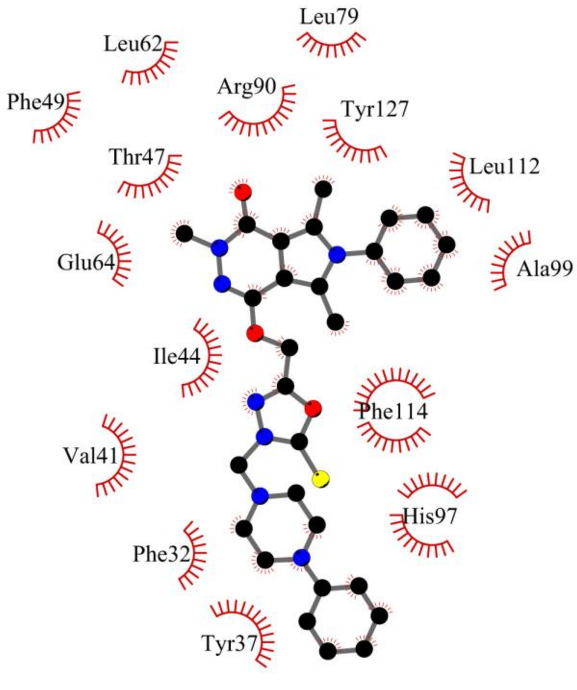

Figure S2c. Hydrophobic interactions between compound 3 and α1-acid glycoprotein.

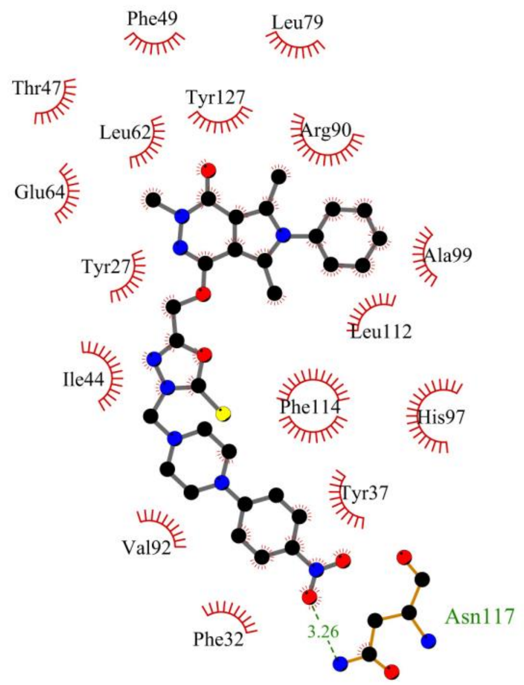

Figure S2d. Interactions between compound 4 and α1-acid glycoprotein.

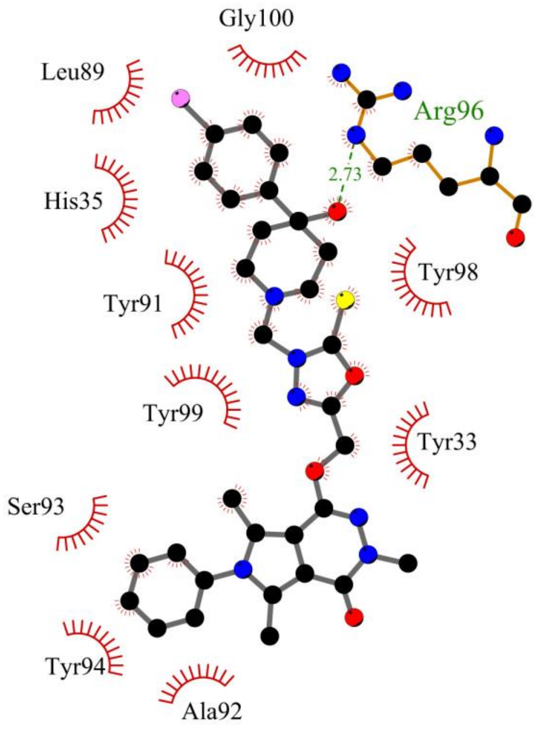

Figure S3a. Interactions between compound 1 and gamma globulin.

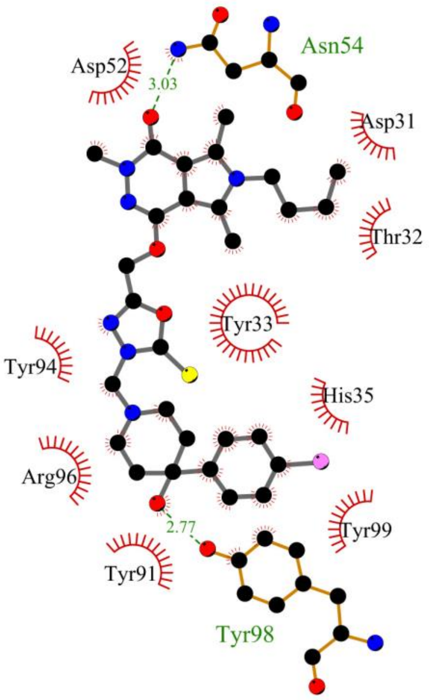

Figure S3b. Interactions between compound 2 and gamma globulin.

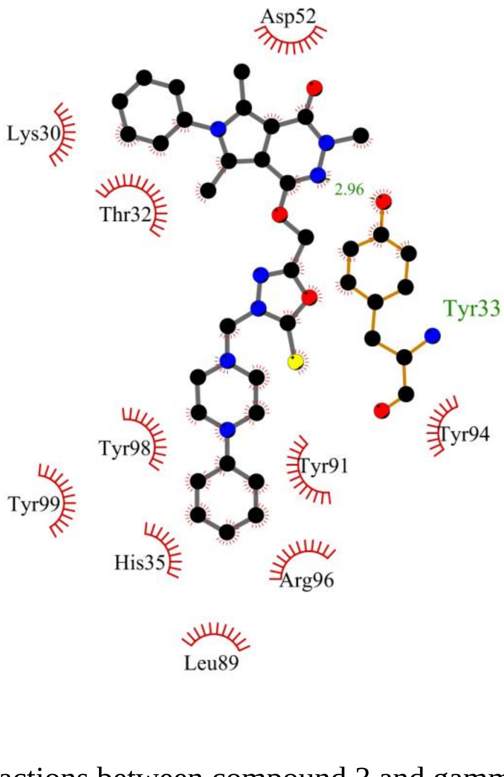

Figure S3c. Interactions between compound 3 and gamma globulin.

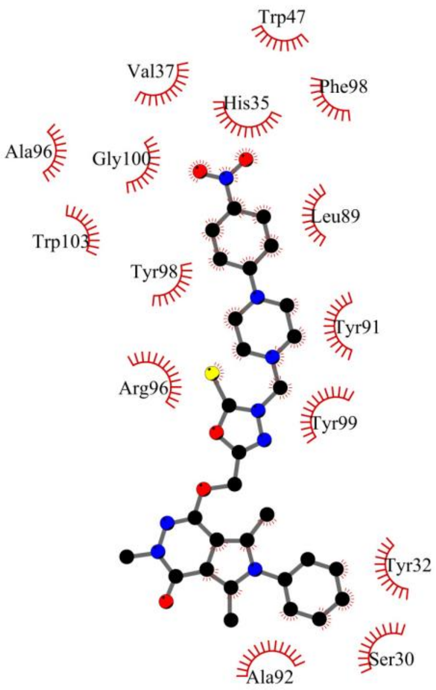

Figure S3d. Interactions between compound 4 and gamma globulin.

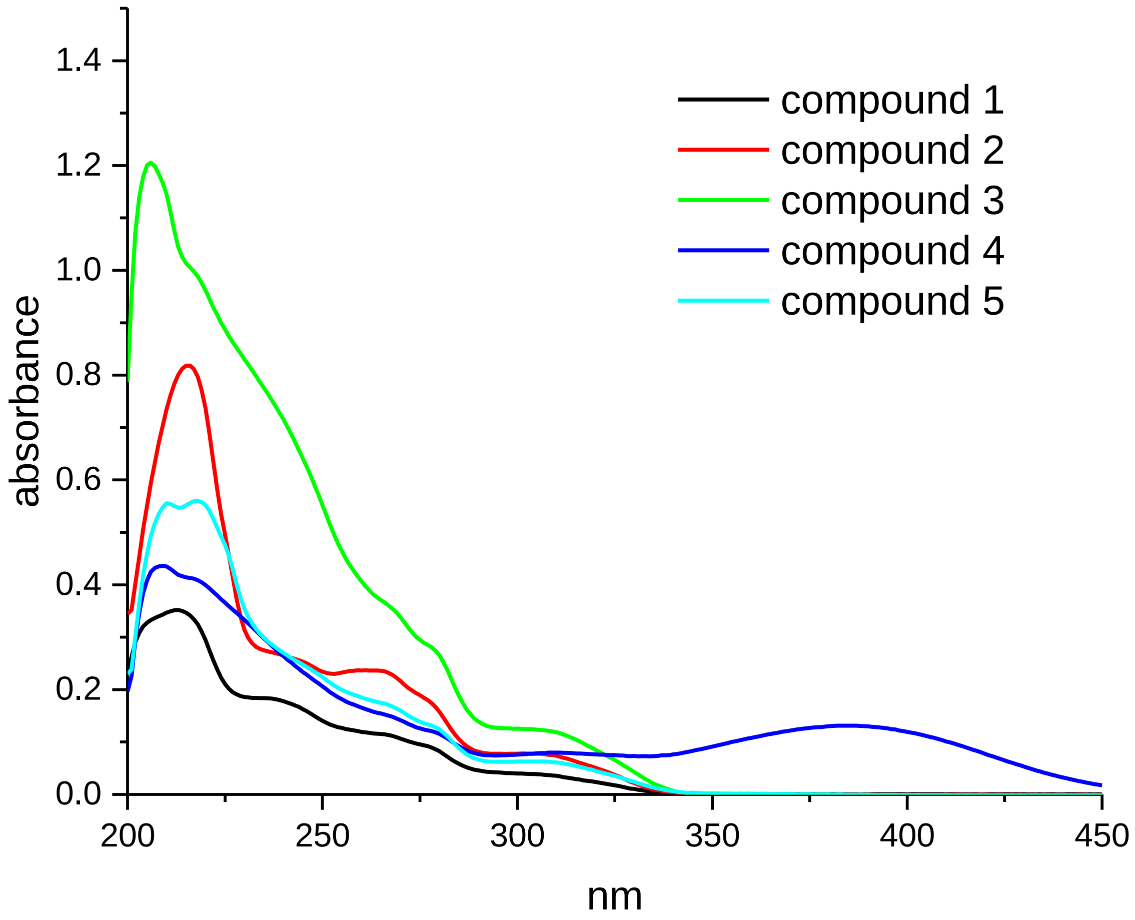

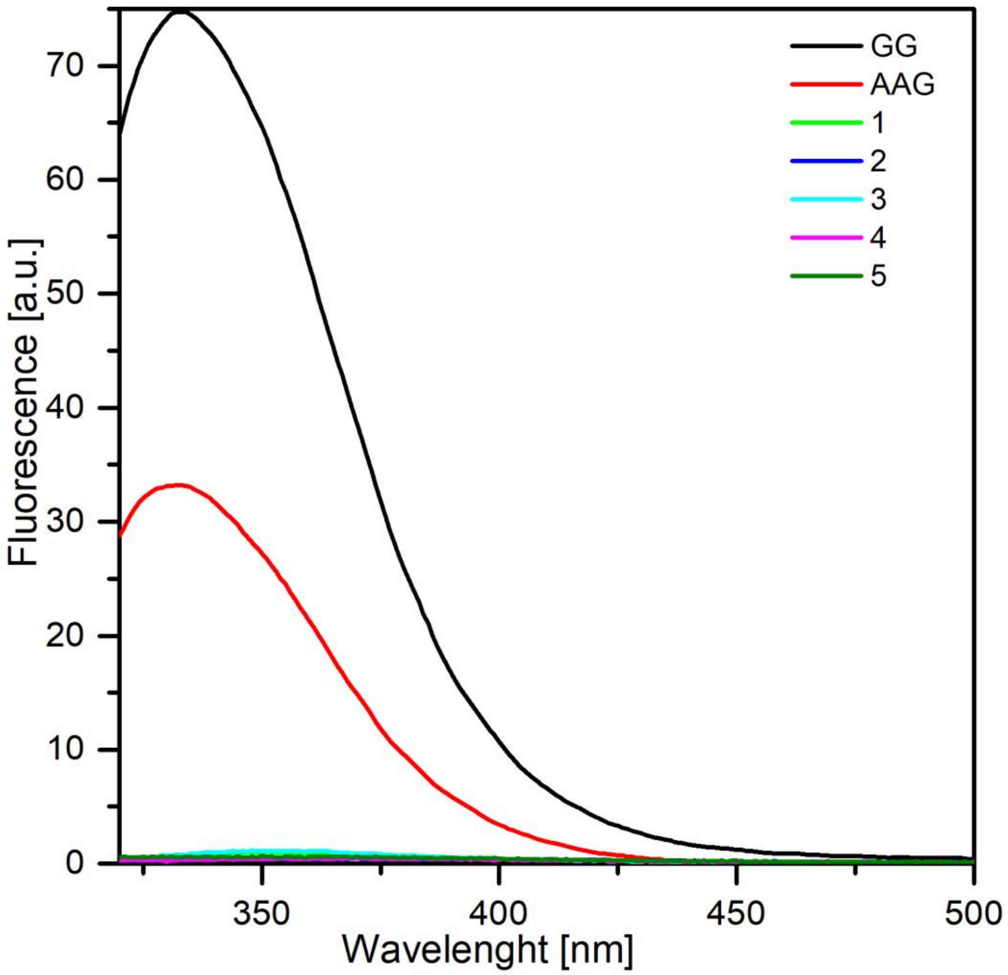

Figure S4. UV-Vis and fluorescence spectra of compounds 1-5. The concentration of compounds was $1 \cdot 10^{-5}$ M and $1 \cdot 10^{-6}$ M for UV-Vis and fluorescence spectroscopy, respectively. The path length in both methods was equal to 10 mm.
